# Supplementary material for: Hospitalization and ambulatory costs related to breast cancer due to physical inactivity in the Brazilian state capitals
Source: PLoS One. 2022 Jan 19;17(1):e0261019. doi: 10.1371/journal.pone.0261019 (PMC8769291; doi:10.1371/journal.pone.0261019)
Supplement: S1 Table — DATASUS (2015, 2016 and 2017). (DOC) [file pone.0261019.s001.doc]

**Supplementary Table 1.** Description of outpatient procedures on breast cancer applied in Brazil. DATASUS (2015, 2016 and 2017).

|  | **English language** | **Portuguese Language** |
| --- | --- | --- |
| Outpatient procedures on breast cancer (code from DATASUS and name) | 0304010413 BREAST RADIOTHERAPY, 0304020133 ADVANCED BREAST CARCINOMA CHEMOTHERAPY -1st LINE, 0304020141 ADVANCED BREAST CARCINOMA CHEMOTHERAPY - 2nd LINE, 0304020338 ADVANCED BREAST CARCINOMA CHEMOTHERAPY -1st LINE, 0304020141 ADVANCED BREAST CARCINOMA CHEMOTHERAPY - 2nd LINE, 0304020338 ADVANCED BREAST CARCINOMA HORMONOTHERAPY 203 1ST LINE ADVANCED BREAST CARCINOMA, 03040 BREAST CANCER HORMONIOTHERAPY POSITIVE RECEPTOR (2nd LINE), 0304020419 HER-2 BREAST CARCINOMA POLYCHEMOTHERAPY POSITIVE – 1st LINE, 0304020427 HER-2 BREAST CARCINOMA MONOCHEMYOTHERAPY POSITIVE – 1st LINE HER-2 POSITIVE HERMONY BREAST CHEMOTHERAPY HER-2 POSITIVE 1st LINE, 0304020443 CHEMOTHERAPY WITH DOUBLE ANTI-HER-2 BREAST CARCINOMA HER-2 POSITIVE – 1st LINE, 0304040029 BREAST CARCINOMA CHEMOTHERAPY (PREVIOUS), 0304040037 BREAST CARCINOMA CHEMOTHERAPY - IN CARCINOMA 2nd LINE, 0304040185 HER-2 POSITIVE BREAST CARCINOMA POLYCHEMOTHERAPY AT STAGE III (PREVIOUS), 0304040193 HORMONIOTHERAPY FOR STAGED BREAST CARCINOMA III (PREVIOUS), 0304050040 HORMONIOTHERAPY OF BREAST CARCINOMA IN STAGE I, 0304050059 TREATMENT OF BREAST CANCER W/ COMMITMENT OF 4 OR MORE LYMPHONODES BY CHEMOTHERAPY - IN PACIE, 0304050067 CHEMOTHERAPY OF CHARCINOMA OF THE III CARCINOMA, 030 IN STAGE BREAST CANCER STAGE II, 0304050083 BREAST CANCER TREATMENT POSITIVE RECEPTOR W/ COMPROMISE OF 4 OR MORE LYMPHONODES PER HORMON, 0304050091 TREATMENT OF BREAST CANCER POSITIVE RECEPTOR W/ COMPROMISE OF UP TO 3 LYMPHONODES PER CHEMOTHERA, 0304050105 MAMAMATATION TREATMENT BY HORMONIOTHERAPY (STAGE II CLINICAL / PATHOLOGICAL), 0304050113 HORMONIOTHERAPY OF BREAST CARCINOMA IN STAGE III, 0304050121 HORMONIOTHERAPY OF BREAST CARCINOMA IN STAGE II, 0304050130 CHEMOTHERAPY OF CHAMINA CARCINOMA I IN STAGE III, 0304050121 BREAST CARCINOMA CHEMOTHERAPY IN STAGE II, 0304050130 CHAMINA CARCINOMA CHEMIOTHERAPY IN STAGE II, 00501 / PATHOLOGICAL WITH POSI, AXILLARY LYMPHONODES, 0304050245 TREATMENT OF BREAST CANCER RECEIVER P OSITIVO WITHOUT IMPAIRMENT OF AXILLARY LYMPHONODES BY CHEMIOT, 0304050261 POLYCHEMOTHERAPY OF HER-2 BREAST CARCINOMA POSITIVE IN STAGE I (ADJUVANT), 0304050270 POLYCHEMOTHERAPY OF BREAST CARCINOMA HER-2 POSITIVE IN STAGE CHARCINOMA II (ADJUVANT), 0304050270 POLYCHEMOTHERAPY OF BREAST CARCINOMA HER-2 POSITIVE IN STAGE CHARCINOMA II (ADJUVANT) 0304050270 HER-2 POSITIVE IN STAGE III (ADJUVANT), 0304050296 MONOCHEMOTHERAPY FOR BREAST CARCINOMA HER-2 POSITIVE IN STAGE I (ADJUVANT), 0304050300 MONOCHEMOTHERAPY FOR BREAST CARCINOMA HER-2 POSITIVE IN STAGE II (ADJUVANT), 03040518 HER-2 POSITIVE BREAST IN STAGE III (ADJUVANT), 0410010057 RADICAL MASTECTOMY W/ LYMPHADENECTOMY, 0410010065 SIMPLE MASTECTOMY, 0410010090 RECONSTRUCTIVE BREAST PLASTIC - POS MASTECTOMY W/ PROSTHESIS IMPLANT, 0410010200 SIMPLE BREAST MASTECTOMY WITH RESTECTOMICAL MASTECTOMY, 0410010200 SIMPLE BREAST MASTECTOMY, RESPOMICS IN ONCOLOGY, 0416120032 SIMPLE MASTECTOMY IN ONCOLOGY | 0304010413 RADIOTERAPIA DE MAMA, 0304020133 QUIMIOTERAPIA DO CARCINOMA DE MAMA AVANÇADO -1ª LINHA, 0304020141 QUIMIOTERAPIA DO CARCINOMA DE MAMA AVANÇADO - 2ª LINHA, 0304020338 HORMONIOTERAPIA DO CARCINOMA DE MAMA AVANÇADO - 2ª LINHA, 0304020346 HORMONIOTERAPIA DO CARCINOMA DE MAMA AVANÇADO- 1ª LINHA, 0304020354 HORMONIOTERAPIA DE CANCER DE MAMA RECEPTOR POSITIVO (2ª LINHA), 0304020419 POLIQUIMIOTERAPIA DO CARCINOMA DE MAMA HER-2 POSITIVO – 1ª LINHA, 0304020427 MONOQUIMIOTERAPIA DO CARCINOMA DE MAMA HER-2 POSITIVO – 1ª LINHA, 0304020435 POLIQUIMIOTERAPIA COM DUPLO ANTI HER-2 DO CARCINOMA DE MAMA HER-2 POSITIVO 1ª LINHA, 0304020443 QUIMIOTERAPIA COM DUPLO ANTI-HER-2 DO CARCINOMA DE MAMA HER-2 POSITIVO – 1ª LINHA, 0304040029 QUIMIOTERAPIA DO CARCINOMA DE MAMA (PRÉVIA), 0304040037 QUIMIOTERAPIA DO CARCINOMA DE MAMA EM ESTÁDIO III - 2 ª LINHA, 0304040185 POLIQUIMIOTERAPIA DO CARCINOMA DE MAMA HER-2 POSITIVO EM ESTÁDIO III (PRÉVIA), 0304040193 HORMONIOTERAPIA DO CARCINOMA DE MAMA EM ESTÁDIO III (PRÉVIA), 0304050040 HORMONIOTERAPIA DO CARCINOMA DE MAMA EM ESTÁDIO I, 0304050059 TRATAMENTO DE CANCER DE MAMA C/ COMPROMETIMENTO DE 4 OU MAIS LINFONODOS POR QUIMIOTERAPIA - EM PACIE, 0304050067 QUIMIOTERAPIA DO CARCINOMA DE MAMA EM ESTÁDIO III, 0304050075 QUIMIOTERAPIA DO CARCINOMA DE MAMA EM ESTÁDIO II, 0304050083 TRATAMENTO DE CANCER DE MAMA RECEPTOR POSITIVO C/ COMPROMETIMENTO DE 4 OU MAIS LINFONODOS POR HORMON, 0304050091 TRATAMENTO DE CANCER DE MAMA RECEPTOR POSITIVO C/ COMPROMETIMENTO DE ATE 3 LINFONODOS POR QUIMIOTERA, 0304050105 TRATAMENTO DE CANCER DE MAMA RECEPTOR POSITIVO POR HORMONIOTERAPIA (ESTADIO II CLINICO / PATOLOGICO), 0304050113 HORMONIOTERAPIA DO CARCINOMA DE MAMA EM ESTÁDIO III, 0304050121 HORMONIOTERAPIA DO CARCINOMA DE MAMA EM ESTÁDIO II, 0304050130 QUIMIOTERAPIA DO CARCINOMA DE MAMA EM ESTÁDIO I, 0304050148 QUIMIOTERAPIA DO CARCINOMA DE MAMA EM ESTÁDIO II CLÍNICO / PATOLÓGICO COM LINFONODOS AXILARES POSI, 0304050245 TRATAMENTO DO CANCER DE MAMA RECEPTOR POSITIVO S/ COMPROMETIMENTO DE LINFONODOS AXILARES POR QUIMIOT, 0304050261 POLIQUIMIOTERAPIA DO CARCINOMA DE MAMA HER-2 POSITIVO EM ESTÁDIO I (ADJUVANTE), 0304050270 POLIQUIMIOTERAPIA DO CARCINOMA DE MAMA HER-2 POSITIVO EM ESTÁDIO II (ADJUVANTE), 0304050288 POLIQUIMIOTERAPIA DO CARCINOMA DE MAMA HER-2 POSITIVO EM ESTÁDIO III (ADJUVANTE), 0304050296 MONOQUIMIOTERAPIA DO CARCINOMA DE MAMA HER-2 POSITIVO EM ESTÁDIO I (ADJUVANTE), 0304050300 MONOQUIMIOTERAPIA DO CARCINOMA DE MAMA HER-2 POSITIVO EM ESTÁDIO II (ADJUVANTE), 0304050318 MONOQUIMIOTERAPIA DO CARCINOMA DE MAMA HER-2 POSITIVO EM ESTÁDIO III (ADJUVANTE), 0410010057 MASTECTOMIA RADICAL C/ LINFADENECTOMIA, 0410010065 MASTECTOMIA SIMPLES, 0410010090 PLASTICA MAMARIA RECONSTRUTIVA - POS MASTECTOMIA C/ IMPLANTE DE PROTESE, 0410010197 MASTECTOMIA SIMPLES BILATERAL SOB PROCESSO TRANSEXUALIZADOR, 0416120024 MASTECTOMIA RADICAL COM LINFADENECTOMIA AXILAR EM ONCOLOGIA, 0416120032 MASTECTOMIA SIMPLES EM ONCOLOGIA |
