# Supplementary material for: Hospitalization and ambulatory costs related to breast cancer due to physical inactivity in the Brazilian state capitals
Source: PLoS One. 2022 Jan 19;17(1):e0261019. doi: 10.1371/journal.pone.0261019 (PMC8769291; doi:10.1371/journal.pone.0261019)
Supplement: S2 Table — *rate per 100,000 inhabitants. Reference population: Reference population: women residing in each capital for each year (aged ≥ 20 years). (DOC) [file pone.0261019.s002.doc]

**Supplementary Table 2.** Incidence of breast cancer among women in Brazilian state capitals and Federal District (aged ≥ 20 years).

|  | **2015** | **2016** | **2017** | **2015** | **2016** | **2017** |
| --- | --- | --- | --- | --- | --- | --- |
|  | **Number** | **Number** | **Number** | **Rate*** | **Rate*** | **Rate*** |
| Aracaju | 31 | 206 | 321 | 12.72 | 82.94 | 126.83 |
| Belém | 326 | 596 | 558 | 58.61 | 105.73 | 97.65 |
| Belo Horizonte | 1,182 | 1,563 | 1,488 | 117.37 | 153.66 | 144.86 |
| Boa Vista | 37 | 44 | 68 | 35.70 | 40.93 | 59.77 |
| Brasília | 423 | 627 | 608 | 39.95 | 57.94 | 55.00 |
| Campo Grande | 323 | 347 | 319 | 101.81 | 107.43 | 97.05 |
| Cuiabá | 275 | 416 | 365 | 127.79 | 190.61 | 164.97 |
| Curitiba | 881 | 920 | 1,028 | 120.18 | 123.80 | 136.52 |
| Florianópolis | 309 | 433 | 464 | 164.91 | 226.10 | 237.30 |
| Fortaleza | 1,612 | 1,816 | 1,639 | 161.44 | 179.34 | 159.64 |
| Goiânia | 726 | 823 | 783 | 131.63 | 146.52 | 136.94 |
| João Pessoa | 633 | 614 | 638 | 209.81 | 200.21 | 204.74 |
| Macapá | 52 | 42 | 49 | 36.10 | 28.21 | 31.87 |
| Maceió | 378 | 457 | 392 | 101.79 | 121.09 | 102.20 |
| Manaus | 353 | 347 | 323 | 52.28 | 50.02 | 45.32 |
| Natal | 686 | 723 | 669 | 205.79 | 213.75 | 194.97 |
| Palmas | 78 | 85 | 84 | 87.32 | 92.02 | 88.03 |
| Porto Alegre | 1,670 | 1,525 | 1,528 | 277.29 | 251.31 | 249.95 |
| Porto Velho | 130 | 160 | 214 | 82.75 | 99.07 | 129.02 |
| Recife | 1,641 | 1,631 | 1,578 | 252.07 | 247.71 | 236.95 |
| Rio Branco | 45 | 88 | 77 | 36.48 | 69.56 | 59.34 |
| Rio de Janeiro | 2,975 | 3,016 | 2,917 | 111.67 | 112.30 | 107.73 |
| Salvador | 1,538 | 1,695 | 1,841 | 136.68 | 148.67 | 159.41 |
| São Luís | 518 | 580 | 512 | 128.07 | 141.45 | 123.11 |
| São Paulo | 4,917 | 4,656 | 4,341 | 105.82 | 99.11 | 91.41 |
| Teresina | 648 | 635 | 543 | 200.89 | 194.68 | 164.63 |
| Vitória | 768 | 738 | 669 | 552.93 | 523.79 | 468.04 |

*rate per 100,000 inhabitants. Reference population: Reference population: women residing in each capital for each year (aged ≥ 20 years).
