# Supplementary material for: Hospitalization and ambulatory costs related to breast cancer due to physical inactivity in the Brazilian state capitals
Source: PLoS One. 2022 Jan 19;17(1):e0261019. doi: 10.1371/journal.pone.0261019 (PMC8769291; doi:10.1371/journal.pone.0261019)
Supplement: S3 Table — *Age-standardized (per 100,000 inhabitants). (DOC) [file pone.0261019.s003.doc]

**Supplementary Table 3.** Number and age-standardized rate of hospitalizations (per 100,000 inhabitants) due to breast cancer in women aged ≥ 20 years in 2015, 2016 and 2017, in Brazil and in the Brazilian state capitals.

|  | **2015** | **2016** | **2017** | **Total** | **2015** | **2016** | **2017** | **Total** |
| --- | --- | --- | --- | --- | --- | --- | --- | --- |
|  | **Number** | **Number** | **Number** | **Number** | **Rate*** | **Rate*** | **Rate*** | **Rate*** |
| Brazil | 17,364 | 17,908 | 18,220 | 53,492 | 92.74 | 94.35 | 94.69 | 93.94 |
| Aracaju | 60 | 82 | 113 | 255 | 24.63 | 33.02 | 44.65 | 34.22 |
| Belém | 199 | 197 | 245 | 641 | 35.78 | 34.95 | 42.88 | 37.90 |
| Belo Horizonte | 1,195 | 1,469 | 1,615 | 4,279 | 118.66 | 144.42 | 157.22 | 140.23 |
| Boa Vista | 55 | 66 | 58 | 179 | 53.06 | 61.39 | 50.98 | 55.09 |
| Brasília | 610 | 696 | 753 | 2,059 | 57.61 | 64.32 | 68.12 | 63.42 |
| Campo Grande | 322 | 372 | 373 | 1,067 | 101.49 | 115.16 | 113.48 | 110.12 |
| Cuiabá | 166 | 183 | 181 | 530 | 77.14 | 83.85 | 81.81 | 80.95 |
| Curitiba | 615 | 743 | 781 | 2,139 | 83.90 | 99.99 | 103.71 | 95.95 |
| Florianópolis | 180 | 218 | 178 | 576 | 96.07 | 113.83 | 91.03 | 100.28 |
| Fortaleza | 792 | 722 | 721 | 2,235 | 79.32 | 71.30 | 70.23 | 73.57 |
| Goiânia | 455 | 645 | 661 | 1,761 | 82.49 | 114.83 | 115.60 | 104.51 |
| João Pessoa | 233 | 227 | 310 | 770 | 77.23 | 74.02 | 99.48 | 83.70 |
| Macapá | 50 | 43 | 60 | 153 | 34.71 | 28.88 | 39.02 | 34.25 |
| Maceió | 253 | 239 | 315 | 807 | 68.13 | 63.33 | 82.12 | 71.27 |
| Manaus | 300 | 288 | 302 | 890 | 44.43 | 41.51 | 42.38 | 42.75 |
| Natal | 377 | 280 | 357 | 1,014 | 113.09 | 82.78 | 104.04 | 99.93 |
| Palmas | 53 | 61 | 48 | 162 | 59.33 | 66.04 | 50.30 | 58.46 |
| Porto Alegre | 791 | 815 | 854 | 2,460 | 131.34 | 134.31 | 139.70 | 135.14 |
| Porto Velho | 150 | 126 | 116 | 392 | 95.49 | 78.02 | 69.94 | 80.92 |
| Recife | 762 | 848 | 863 | 2,473 | 117.05 | 128.79 | 129.58 | 125.19 |
| Rio Branco | 58 | 53 | 66 | 177 | 47.02 | 41.89 | 50.87 | 46.62 |
| Rio de Janeiro | 2,859 | 2,927 | 2,730 | 8,516 | 107.32 | 108.98 | 100.82 | 105.69 |
| Salvador | 1,400 | 1,426 | 1,304 | 4,130 | 124.42 | 125.07 | 112.91 | 120.75 |
| São Luís | 323 | 366 | 378 | 1,067 | 79.86 | 89.26 | 90.89 | 86.72 |
| São Paulo | 4,607 | 4,328 | 4,377 | 13,312 | 99.14 | 92.13 | 92.16 | 94.45 |
| Teresina | 292 | 302 | 282 | 876 | 90.53 | 92.59 | 85.50 | 89.52 |
| Vitória | 207 | 186 | 179 | 572 | 149.03 | 132.01 | 125.23 | 135.31 |

*Age-standardized (per 100,000 inhabitants).
