# Supplementary material for: Hospitalization and ambulatory costs related to breast cancer due to physical inactivity in the Brazilian state capitals
Source: PLoS One. 2022 Jan 19;17(1):e0261019. doi: 10.1371/journal.pone.0261019 (PMC8769291; doi:10.1371/journal.pone.0261019)
Supplement: Attachment — Submitted filename: PONE-D-21-09895_reviewer_19.05.21 to send.pdf [file pone.0261019.s004.pdf]

PLOS ONE

# The economic burden of breast cancer due to physical inactivity in Brazil

--Manuscript Draft--

| | |
|---|---|
| Manuscript Number: | PONE-D-21-09895 |
| Article Type: | Research Article |
| Full Title: | The economic burden of breast cancer due to physical inactivity in Brazil |
| Short Title: | Breast cancer and physical inactivity in Brazil |
| Corresponding Author: | Diego Augusto Santos Silva, Ph.D.<br>Federal University of Santa Catarina<br>Florianópolis, Santa Catarina BRAZIL |
| Keywords: | Epidemiology; Physical activity; Prevention; Brazil; Global Health |
| Abstract: | The aim of this study was to estimate the economic burden of breast cancer due to physical inactivity in the Brazilian female population over a three-year period (2015 to 2017) and to relate these costs to conditions of access and quality of health services in Brazilian states. This study was carried out with data from the Brazilian health system and had as metrics incidence of breast cancer, total and standardized rate hospitalizations by breast cancer, hospitalization costs by breast cancer and prevalence of physical inactivity. The access and quality of health services in each state of Brazil was also analyzed. The Population Attributable Fraction calculation was used. During three-year period the total hospitalization cost related to breast cancer due to physical inactivity in women aged 20-59 years was US$ 3,224,043.20 and in women aged ≥60 years was US$ 4,260,623.66. Both in women aged 20-59 years (rho = .37, p = .04) and in those aged ≥60 years (rho = .43, p = .02), the mean hospitalization cost due to physical inactivity was higher in states with better quality and access to health services. Physical inactivity has contributed to the high number of hospitalizations for breast cancer in Brazil, which resulted in economic burden for health services of more than seven million dollars from 2015 to 2017. |
| Order of Authors: | Diego Augusto Santos Silva, Ph.D. |
| Additional Information: | |
| **Question** | **Response** |
| **Financial Disclosure**<br><br>Enter a financial disclosure statement that describes the sources of funding for the work included in this submission. Review the submission guidelines for detailed requirements. View published research articles from *PLOS ONE* for specific examples.<br><br>This statement is required for submission and **will appear in the published article** if the submission is accepted. Please make sure it is accurate. | The author(s) received no specific funding for this work. |

*Powered by Editorial Manager® and ProduXion Manager® from Aries Systems Corporation*

| | |
|---|---|
| **Unfunded studies**<br>Enter: *The author(s) received no specific funding for this work.*<br><br>**Funded studies**<br>Enter a statement with the following details:<br>• Initials of the authors who received each award<br>• Grant numbers awarded to each author<br>• The full name of each funder<br>• URL of each funder website<br>• Did the sponsors or funders play any role in the study design, data collection and analysis, decision to publish, or preparation of the manuscript?<br>• **NO** - Include this sentence at the end of your statement: *The funders had no role in study design, data collection and analysis, decision to publish, or preparation of the manuscript.*<br>• **YES** - Specify the role(s) played.<br><br>* typeset | |
| **Competing Interests**<br><br>Use the instructions below to enter a competing interest statement for this submission. On behalf of all authors, disclose any competing interests that could be perceived to bias this work—acknowledging all financial support and any other relevant financial or non-financial competing interests.<br><br>This statement is required for submission and **will appear in the published article** if the submission is accepted. Please make sure it is accurate and that any funding sources listed in your Funding Information later in the submission form are also declared in your Financial Disclosure statement.<br><br>View published research articles from *PLOS ONE* for specific examples. | The authors have declared that no competing interests exist. |

| | |
|---|---|
| **NO authors have competing interests**<br><br>Enter: *The authors have declared that no competing interests exist.*<br><br>**Authors with competing interests**<br><br>Enter competing interest details beginning with this statement:<br><br>*I have read the journal's policy and the authors of this manuscript have the following competing interests: [insert competing interests here]*<br><br>* typeset | |
| **Ethics Statement**<br><br>Enter an ethics statement for this submission. This statement is required if the study involved:<br><br>• Human participants<br>• Human specimens or tissue<br>• Vertebrate animals or cephalopods<br>• Vertebrate embryos or tissues<br>• Field research<br><br>Write "N/A" if the submission does not require an ethics statement.<br><br>General guidance is provided below. Consult the submission guidelines for detailed instructions. **Make sure that all information entered here is included in the Methods section of the manuscript.** | The study protocol was approved by National Ethics Committee on Research with Human Beings (CONEP/BRAZIL), and has been conducted in full accordance with ethical principles, including provisions of the World Medical Association Declaration of Helsinki (Ethical Application Ref: 65610017.1.0000.0008). |

Powered by Editorial Manager® and ProduXion Manager® from Aries Systems Corporation

| | |
|---|---|
| **Format for specific study types**<br><br>**Human Subject Research (involving human participants and/or tissue)**<br>• Give the name of the institutional review board or ethics committee that approved the study<br>• Include the approval number and/or a statement indicating approval of this research<br>• Indicate the form of consent obtained (written/oral) or the reason that consent was not obtained (e.g. the data were analyzed anonymously)<br><br>**Animal Research (involving vertebrate animals, embryos or tissues)**<br>• Provide the name of the Institutional Animal Care and Use Committee (IACUC) or other relevant ethics board that reviewed the study protocol, and indicate whether they approved this research or granted a formal waiver of ethical approval<br>• Include an approval number if one was obtained<br>• If the study involved *non-human primates*, add *additional details* about animal welfare and steps taken to ameliorate suffering<br>• If anesthesia, euthanasia, or any kind of animal sacrifice is part of the study, include briefly which substances and/or methods were applied<br><br>**Field Research**<br><br>Include the following details if this study involves the collection of plant, animal, or other materials from a natural setting:<br>• Field permit number<br>• Name of the institution or relevant body that granted permission | |
| **Data Availability**<br><br>Authors are required to make all data underlying the findings described fully available, without restriction, and from the time of publication. PLOS allows rare exceptions to address legal and ethical concerns. See the PLOS Data Policy and FAQ for detailed information. | Yes - all data are fully available without restriction |

Powered by Editorial Manager® and ProduXion Manager® from Aries Systems Corporation

| | |
|---|---|
| A Data Availability Statement describing where the data can be found is required at submission. Your answers to this question constitute the Data Availability Statement and **will be published in the article**, if accepted.<br><br>**Important:** Stating 'data available on request from the author' is not sufficient. If your data are only available upon request, select 'No' for the first question and explain your exceptional situation in the text box.<br><br>Do the authors confirm that all data underlying the findings described in their manuscript are fully available without restriction? | |
| **Describe where the data may be found in full sentences. If you are copying our sample text, replace any instances of XXX with the appropriate details.**<br><br>• If the data are **held or will be held in a public repository**, include URLs, accession numbers or DOIs. If this information will only be available after acceptance, indicate this by ticking the box below. For example: *All XXX files are available from the XXX database (accession number(s) XXX, XXX.).*<br>• If the data are all contained **within the manuscript and/or Supporting Information files**, enter the following: *All relevant data are within the manuscript and its Supporting Information files.*<br>• If neither of these applies but you are able to provide **details of access elsewhere**, with or without limitations, please do so. For example:<br><br>*Data cannot be shared publicly because of [XXX]. Data are available from the XXX Institutional Data Access / Ethics Committee (contact via XXX) for researchers who meet the criteria for access to confidential data.*<br><br>*The data underlying the results presented in the study are available from (include the name of the third party* | All data are publicly available by Brazilian Unified Health System accessed via Department of Informatics (DATASUS) of free access and charge: https://datasus.saude.gov.br/informacoes-de-saude-tabnet/ |

| | |
|---|---|
| *and contact information or URL).*<br>• This text is appropriate if the data are owned by a third party and authors do not have permission to share the data.<br><br>* typeset | |
| Additional data availability information: | |

Manuscript Click here to access/download;Manuscript;Article.doc

# The economic burden of breast cancer due to physical inactivity in Brazil

Diego Augusto Santos Silva[1*¶]

1. Research Center in Kinanthropometry and Human Performance, Sports Center, Federal University of Santa Catarina, Florianopolis, Brazil.

*Corresponding author

Email: diegoaugustoss@yahoo.com.br

¶ These authors contributed equally to this work.

# Abstract

The aim of this study was to estimate the economic burden of breast cancer due to physical inactivity in the Brazilian female population over a three-year period (2015 to 2017) and to relate these costs to conditions of access and quality of health services in Brazilian states. This study was carried out with data from the Brazilian health system and had as metrics incidence of breast cancer, total and standardized rate hospitalizations by breast cancer, hospitalization costs by breast cancer and prevalence of physical inactivity. The access and quality of health services in each state of Brazil was also analyzed. The Population Attributable Fraction calculation was used. During three-year period the total hospitalization cost related to breast cancer due to physical inactivity in women aged 20-59 years was US$ 3,224,043.20 and in women aged ≥60 years was US$ 4,260,623.66. Both in women aged 20-59 years (rho = .37, p = .04) and in those aged ≥60 years (rho = .43, p = .02), the mean hospitalization cost due to physical inactivity was higher in states with better quality and access to health services. Physical inactivity has contributed to the high number of hospitalizations for breast cancer in Brazil, which resulted in economic burden for health services of more than seven million dollars from 2015 to 2017.

*Keywords:* Epidemiology, Physical activity, Prevention, Brazil, Global Health.

# Introduction

Breast cancer is the type of cancer most commonly diagnosed in the female population and in 2015, it accounted for 523,000 deaths worldwide [1, 2]. Breast cancer has multifactorial cause in which genetic and lifestyle aspects stand out [1]. Among modifiable lifestyle aspects, physical inactivity plays an important role in the prevention and treatment of this neoplasm because the regular practice of physical exercise is associated with lower concentrations of female sex hormones and lower levels of body fat [1]. Both factors (high concentrations of female sex hormones and high levels of body fat) are associated with increased risk of breast cancer, especially in postmenopausal women [1, 2]. Epidemiological surveys on breast cancer mortality due to physical inactivity estimated 29,605 deaths in 1990 and 46,720 deaths in 2015 around the world [3].

In addition to mortality estimates presented in literature, the analysis of the economic burden of breast cancer can serve to assess public policies and for governments to prioritize preventive measures in order to reduce the economic cost of this disease [4]. Data from Spain published in 2020 reported that the total cost of breast cancer in that country over a five-year period was € 469.92,731 [4]. In Mexico, cost projection of US$ 245 million was estimated for procedures related to breast cancer throughout the lives of women born in 2012 [5]. In the United States of America, 2007 data have shown total cost of US$ 12.2 billion with procedures related to breast cancer [6]. In Brazil, cost of approximately US$ 5.8 million was estimated in 2013 for hospitalizations due to breast cancer [7].

In addition to information on the global economic burden of the disease, which can be analyzed by metrics related to cost related to hospitalizations, medicines and procedures in general [6, 7], studies have prioritized making estimates by risk factors

[8]. These risk factor estimates allow managers and society to become aware of how much each action in a specific risk factor would result in savings. A systematic review developed with studies published until 2014 found 24 articles that estimated the cost of physical inactivity related to various non-communicable diseases and reported that physical inactivity had high economic impact for the health sector, regardless of economic analysis metric [8]. Ding et al. [9] estimated costs of physical inactivity related to various non-communicable diseases around the world and found that in 2013, this cost was US$ 53.8 billion, of which US$ 31.2 billion were paid by the public sector. In relation to breast cancer, a survey highlighted the impact of physical inactivity on hospitalizations for breast cancer and estimated an approximate expenditure of US$ 1.2 million in a single country [7].

Brazil has continental dimensions, with evident social and economic discrepancies among states [3, 10]. These discrepancies are reflected in living conditions, lifestyles and in the quality and access to health services [10], which, in turn, reflect in the amount of care for breast cancer and the cost of each state with the treatment of the disease [3]. Thus, the estimate of the economic burden of breast cancer due to physical inactivity by Brazilian state can provide information on how each state has faced the problem of breast cancer related to physical inactivity and also can provide information on the health inequality in Brazil.

The aim of this study was to estimate the economic burden of breast cancer due to physical inactivity in the female population of Brazilian states over a three-year period (2015 to 2017) and to relate these costs to conditions o[illegible]ccess and quality of health services in Brazilian states.

## Materials and methods

## Overview

This descriptive analytical study was carried out with data from the Brazilian Unified Health System accessed via Department of Informatics (DATASUS) of free access and charge. For all Brazilian states, the quality of data from DATASUS is considered high and close to high-income countries [11, 12]. All DATASUS information follows the ethical precepts of the Declaration of Helsinki, so that users' anonymity is respected. This study used information collected by the Ministry of Health of Brazil entitled “Surveillance System of Risk and Protection Factors of Noncommunicable Disease by Telephone Survey - VIGITEL”. This system aims to monitor health indicators of the Brazilian population aged ≥ 18 years through telephone survey and was carried out in 26 Brazilian capitals and the Federal District. Free and informed consent was obtained from all participants at the time of data collection. The VIGITEL study in Brazil protocol was approved by National Ethics Committee on Research with Human Beings (CONEP/BRAZIL), and has been conducted in full accordance with ethical principles, including provisions of the World Medical Association Declaration of Helsinki (Ethical Application Ref: 65610017.1.0000.0008).

## Estimation of incidence, hospitalizations and costs related to breast cancer

The present study examined data from the Brazilian female population in the years 2015, 2016 and 2017 regarding the incidence of breast cancer, number of hospitalizations and costs related to breast cancer in all Brazilian states. For the present study, malignant breast cancer was characterized according to the latest

International Classification of Diseases, 10th Revision (ICD-10). ICD-10 categories for malignant breast cancer analyzed were C50, C50.0, C50.1, C50.2, C50.3, C50.4, C50.5, C50.6, C50.8, C50.9 [13].

Information regarding the incidence of breast cancer in the Brazilian female population aged 20 years and over in years 2015, 2016 and 2017 was taken from estimates of the Global Burden of Disease [2], which analyzes estimates and makes information available free of charge based on data provided by countries. Such information is used by the Brazilian government for the epidemiological surveillance of cancer cases [3].

From the access to the DATASUS system, information from years 2015, 2016 and 2017 on the total number of hospitalizations and the cost of these hospitalizations in the Brazilian female population aged 20-59 years and ≥60 years was extracted [14]. This age stratification was chosen because in middle-income countries, from the age of 60, the population is considered elderly. Thus, the present study proposes estimates according to age group, as there are different health policies in each age group for adults and for the elderly [15]. From values of years 2015, 2016 and 2017, the mean cost of hospitalizations in these three years (with standard deviation estimate) and the total cost were calculated from the sum of annual values. For international comparison, estimates in reais (R$ - Brazilian currency) were converted into US dollars (US$). For this purpose, the mean value of the dollar quotation in the three years of the survey was considered (US$ 1.0 = R$ 3,33) [16].

## Estimated prevalence of physical inactivity

For estimates on the prevalence of physical inactivity in women aged 20-59 years and ≥60 years, national surveys of the Surveillance System for Risk and Protective Factors for Chronic Diseases by Telephone Survey (VIGITEL) for years 2015, 2016 and 2017 were used [17-19]. Such a system was implemented in Brazil in 2006 and is consolidated as a health surveillance and management system. The sampling procedures used in this survey aim to obtain, in each capital of the 26 Brazilian states and the Federal District, probabilistic samples of the population of adults living in households served by at least one fixed telephone line. The system establishes minimum sample size of approximately 2 thousand individuals in each city to estimate, with 95% confidence coefficient and maximum error of two percentage points, the frequency of any risk factor in the adult population. Maximum errors of three percentage points are expected for specific estimates, according to sex, assuming similar proportions of men and women in the sample [17-19]. Smaller samples are accepted in locations where fixed telephone coverage is less than 40% of households and where the absolute number of households with a telephone line is less than 50 thousand. In this case, estimates for the adult population will have maximum error of three percentage points, being of four percentage points the same error for sex-specific estimates [17-19].

In 2015, 54,174 individuals were evaluated, of which 33,806 were women (n = 730 women aged 18-19 years; n = 20,339 women aged 20-59 years; n = 12,737 women aged ≥60 years). In 2016, 53,210 individuals were evaluated, 32,952 of which were women (n = 757 women aged 18-19 years; n = 19,539 women aged 20-59 years; n = 12,656 women aged ≥60 years). In 2017, 53,034 individuals were evaluated, of which 33,530 were women (n = 674 women aged 18-19 years; n = 18.404 women aged 20-59 years; n = 14,452 women aged ≥60 years).

In the present study, estimates of physical inactivity were considered in all domains (leisure, transport, occupation and domestic activities). Such information is obtained through standardized questionnaire validated for the Brazilian population [20, 21]. Physically inactive were subjects who did not practice any free-time physical activity in the last three months of the interview and those who did not make intense physical efforts at work, did not commute to work or school and were not responsible for heavy cleaning of the house, as recommended in literature [22]. For these estimates, sampling weight and sampling strata were considered.

## Analytical approach

Information on the breast cancer was presented in absolute terms and at standardized rates by age. For the calculation of rates, the most current data from estimates of the female population aged 20-59 years and ≥ 60 years in each of the Brazilian states in 2015, 2016 and 2017 [23] were used. Rates were presented for a population of 100,000 inhabitants.

To estimate the cost of hospitalizations for breast cancer due to physical inactivity, the Population Attributable Fraction (PAF) calculation strategy was used, which is also used in studies on the global burden of diseases attributable to risk factors [2, 3] and in other studies that estimate the cost of physical inactivity [7-9]; This metric identifies the percentage reduction in the disease, in a given year, if the risk factor (physical inactivity) was not present. That is, if the population met the guidelines for physical activity [22]. For this, the following equation was used: $PAF = \{[p * (RR - 1)] / [p * (RR - 1) + 1]\}$, where ‘p’ refers to the prevalence of exposure, ‘RR’ refers to the relative risk attributed to the exposure. RR of the relationship between physical activity and breast cancer was obtained from meta-analysis that analyzed

the dose-response between these variables with the inclusion of adjustment covariates based on studies with good methodological quality [24]. RR considered was 1.40, which indicated the risk of breast cancer in women who did not meet physical activity recommendations of 600 METs minutes/week [22].

The present study also used the Healthcare Access and Quality (HAQ) Index for each of the Brazilian states [25]. This index ranges from zero to 100, in which the higher the value, the better the indicators of access and quality of health service in the evaluated location. The construction of this index takes into account 32 different causes of mortality that are amenable to medical care, which allows comparing the different locations [25, 26]. Many studies have used this index to assess the relationship between health outcomes and quality and access to health services [25-28].

## Results

Breast cancer incidence in Brazilian women in 2015 was 48,254 cases, 50,749 cases in 2016, and 52,607 cases in 2017. These values represented incidence rate (per 100,000 inhabitants) of 40.3 in 2015, 41.3 in 2016 and 41.7 in 2017. S1 Table shows information on the incidence of breast cancer in women aged ≥ 20 years in years 2015, 2016 and 2017 in all Brazilian states.

In 2015, Brazil registered 37,173 hospitalizations by breast cancer in women aged 20-59 years, which represented a standardized hospitalization rate (per 100,000 inhabitants) of 63.5. In 2016, Brazil registered 39,230 hospitalizations by breast cancer in women aged 20-59 years, which represented a standardized hospitalization rate of 66.3 (per 100,000 inhabitants). In 2017, Brazil registered 40,583 hospitalizations by breast cancer in women aged 20-59 years, which

represented a standardized hospitalization rate of 67.9 (per 100,000 inhabitants). Table 1 shows this information in all Brazilian states.

**Table 1. Number and age-standardized of hospitalizations (per 100,000 inhabitants) due to breast cancer in women aged 20 to 59 in 2015, 2016 and 2017, in Brazil and in the states.**

| | 2015 | 2016 | 2017 | Total | 2015 | 2016 | 2017 | Total |
|---|---|---|---|---|---|---|---|---|
| | Number | Number | Number | Number | Rate* | Rate* | Rate* | Rate* |
| Brazil | [illegible] | 39,230 | 40,583 | 116,986 | 63.5 | 66.3 | 67.9 | 65.9 |
| Acre | 47 | 48 | 54 | 149 | 23.4 | 23.3 | 25.5 | 24.1 |
| Alagoas | 379 | 423 | 521 | 1,323 | 41.0 | 45.2 | 54.9 | 47.1 |
| Amapá | 50 | 40 | 42 | 132 | 25.8 | 20.0 | 20.3 | 22.0 |
| Amazonas | 250 | 264 | 239 | 753 | 24.5 | 25.2 | 22.2 | 24.0 |
| Bahia | 2,413 | 2,578 | 2,432 | 7,423 | 55.8 | 59.0 | 55.1 | 56.6 |
| Ceará | 1,194 | 1,195 | 1,263 | 3,652 | 47.6 | 47.0 | 49.0 | 47.9 |
| Distrito Federal | 498 | 566 | 642 | 1,706 | 52.9 | 58.6 | 64.8 | 58.9 |
| Espírito Santo | 1,152 | 1,067 | 1,122 | 3,341 | 101.4 | 92.8 | 96.6 | 96.9 |
| Goiás | 763 | 1,107 | 1,159 | 3,029 | 39.4 | 56.4 | 58.1 | 51.4 |
| Maranhão | 524 | 562 | 560 | 1,646 | 29.0 | 30.6 | 30.1 | 29.9 |
| Mato Grosso | 387 | 485 | 520 | 1,392 | 42.1 | 51.9 | 54.8 | 49.7 |
| Mato Grosso do Sul | 433 | 429 | 447 | 1,309 | 57.8 | 56.4 | 58.0 | 57.4 |
| Minas Gerais | 4,483 | 4,862 | 5,070 | 14,415 | 74.5 | 80.2 | 83.1 | 79.3 |
| Pará | 357 | 369 | 519 | 1,245 | 16.7 | 16.9 | 23.3 | 19.0 |
| Paraíba | 496 | 489 | 620 | 1,605 | 43.8 | 42.7 | 53.6 | 46.8 |
| Paraná | 2,552 | 2,750 | 2,916 | 8,218 | 78.5 | 83.9 | 88.2 | 83.6 |
| Pernambuco | 1,970 | 2,251 | 2,280 | 6,501 | 73.5 | 83.0 | 83.2 | 79.9 |
| Piauí | 481 | 550 | 515 | 1,546 | 53.5 | 60.7 | 56.5 | 56.9 |
| Rio de Janeiro | 3,554 | 3,629 | 3,781 | 10,964 | 73.3 | 74.5 | 77.3 | 75.1 |
| Rio Grande do Norte | 704 | 622 | 703 | 2,029 | 72.0 | 62.7 | 69.9 | 68.2 |
| Rio Grande do Sul | 2,834 | 2,646 | 2,856 | 8,336 | 87.6 | 81.6 | 88.0 | 85.7 |

| | | | | | | | | |
|---|---|---|---|---|---|---|---|---|
| Rondônia | 196 | 263 | 270 | 729 | 39.1 | 51.5 | 52.0 | 47.6 |
| Roraima | 44 | 51 | 55 | 150 | 34.1 | 38.5 | 40.4 | 37.7 |
| Santa Catarina | 1,718 | 1,908 | 1,865 | 5,491 | 86.3 | 94.5 | 91.1 | 90.7 |
| São Paulo | 9,387 | 9,649 | 9,760 | 28,796 | 71.8 | 73.3 | 73.7 | 73.0 |
| Sergipe | 137 | 198 | 227 | 562 | 21.3 | 30.3 | 34.2 | 28.7 |
| Tocantins | 170 | 229 | 145 | 544 | 42.0 | 55.5 | 34.4 | 43.9 |

*Age-standardized (per 100,000 inhabitants).

In 2015, Brazil registered 21,482 hospitalizations by breast cancer in women aged ≥60 years, which represented a standardized hospitalization rate (per 100,000 inhabitants) of 160.9. In 2016, Brazil recorded 22,146 hospitalizations by breast cancer in women aged ≥60 years, which represented a standardized hospitalization rate of 159.3 (per 100,000 inhabitants). In 2017, Brazil registered 23,481 hospitalizations by breast cancer in women aged ≥60 years, which represented standardized hospitalization rate of 162.3 (per 100,000 inhabitants). Table 2 shows this information in all Brazilian states.

**Table 2. Number and age-standardized rate of hospitalizations (per 100,000 inhabitants) due to breast cancer in women ≥ 60 years of age in 2015, 2016 and 2017, in Brazil and in the states.**

| | 2015 | 2016 | 2017 | Total | 2015 | 2016 | 2017 | Total |
|---|---|---|---|---|---|---|---|---|
| | Number | Number | Number | Number | Rate* | Rate* | Rate* | Rate* |
| Brazil | 21,482 | 22,146 | 23,481 | 67,109 | 160.9 | 159.3 | 162.3 | 160.9 |
| Acre | 09 | 21 | 15 | 45 | 33.5 | 74.3 | 50.5 | 53.0 |
| Alagoas | 177 | 169 | 236 | 582 | 102.6 | 94.5 | 127.2 | 108.4 |
| Amapá | 18 | 07 | 19 | 44 | 85.8 | 31.4 | 80.1 | 65.7 |
| Amazonas | 88 | 91 | 116 | 295 | 70.4 | 69.2 | 83.8 | 74.7 |
| Bahia | 1,064 | 1,041 | 1,076 | 3,181 | 118.3 | 111.7 | 111.4 | 113.7 |
| Ceará | 493 | 489 | 583 | 1,565 | 92.3 | 88.8 | 102.8 | 94.7 |

| | | | | | | | | |
|---|---|---|---|---|---|---|---|---|
| Distrito Federal | 226 | 286 | 266 | 778 | 142.3 | 168.9 | 147.6 | 153.0 |
| Espírito Santo | 582 | 603 | 583 | 1,768 | 234.0 | 230.7 | 212.4 | 225.4 |
| Goiás | 349 | 443 | 474 | 1,266 | 98.5 | 119.1 | 121.5 | 113.4 |
| Maranhão | 217 | 245 | 294 | 756 | 69.2 | 75.7 | 87.9 | 77.8 |
| Mato Grosso | 191 | 174 | 208 | 573 | 132.6 | 114.2 | 129.1 | 125.3 |
| Mato Grosso do Sul | 245 | 237 | 270 | 752 | 164.3 | 151.4 | 164.3 | 160.0 |
| Minas Gerais | 2,558 | 2,876 | 3,127 | 8,561 | 172.5 | 186.4 | 194.9 | 184.9 |
| Pará | 172 | 171 | 214 | 557 | 54.8 | 52.1 | 62.3 | 56.5 |
| Paraíba | 290 | 338 | 333 | 961 | 111.0 | 126.1 | 121.1 | 119.5 |
| Paraná | 1,454 | 1,608 | 1,682 | 4,744 | 188.8 | 199.9 | 200.2 | 196.5 |
| Pernambuco | 1,074 | 1,081 | 1,229 | 3,384 | 184.3 | 179.6 | 197.6 | 187.3 |
| Piauí | 244 | 298 | 226 | 768 | 131.9 | 155.9 | 114.4 | 133.9 |
| Rio de Janeiro | 2,580 | 2,620 | 2,701 | 7,901 | 181.8 | 178.1 | 177.1 | 178.9 |
| Rio Grande do Norte | 348 | 319 | 364 | 1,031 | 164.8 | 146.1 | 161.2 | 157.4 |
| Rio Grande do Sul | 1,939 | 1,915 | 1,925 | 5,779 | 194.9 | 185.6 | 179.9 | 186.6 |
| Rondônia | 109 | 105 | 103 | 317 | 164.8 | 150.4 | 139.9 | 151.3 |
| Roraima | 15 | 18 | 15 | 48 | 111.3 | 125.0 | 97.5 | 110.9 |
| Santa Catarina | 819 | 899 | 998 | 2,716 | 180.4 | 188.5 | 199.4 | 189.8 |
| São Paulo | 6,101 | 5,931 | 6,259 | 18,291 | 186.6 | 173.8 | 175.7 | 178.5 |
| Sergipe | 70 | 76 | 83 | 229 | 60.6 | 63.3 | 66.6 | 63.6 |
| Tocantins | 50 | 85 | 82 | 217 | 77.6 | 126.2 | 116.4 | 107.3 |

*Age-standardized (per 100,000 inhabitants).

The prevalence of physical inactivity in 2015 among Brazilian women aged 20-59 years was 11.6%, 11.0% in 2016, and 10.7% in 2017. In women aged ≥60 years, the prevalence of physical inactivity was 32.1% in 2015, 30.1% in 2016 and 29.6% in 2017. Table 3 shows this information according to each Brazilian state.

**Table 3. Prevalence of physical inactivity in women aged 20-59 years and ≥60 years in 2015, 2016 and 2017, in Brazil and in the states.**

| | Prevalence of physical inactivity | | | | | | | |
|---|---|---|---|---|---|---|---|---|
| | 2015 | | 2016 | | 2017 | | Total | |
| | Aged | | Aged | | Aged | | Aged | |
| | 20-59 | ≥ 60 | 20-59 | ≥ 60 | 20-59 | ≥ 60 | 20-59 | ≥ 60 |
| | % | % | % | % | % | % | % | % |
| Brazil | 11.6 | 32.1 | 11.0 | 30.1 | 10.7 | 29.6 | 11.1 | 30.6 |
| Acre | 12.5 | 38.0 | 10.3 | 33.3 | 10.6 | 34.0 | 11.1 | 35.1 |
| Alagoas | 11.7 | 36.6 | 12.5 | 36.7 | 14.2 | 34.1 | 12.8 | 35.8 |
| Amapá | 11.9 | 37.6 | 11.4 | 33.0 | 11.1 | 30.4 | 11.5 | 33.7 |
| Amazonas | 10.5 | 29.7 | 11.7 | 30.8 | 11.0 | 25.5 | 11.1 | 28.7 |
| Bahia | 10.8 | 34.8 | 10.3 | 31.1 | 8.3 | 31.3 | 9.8 | 32.4 |
| Ceará | 13.1 | 32.6 | 12.1 | 32.8 | 10.7 | 33.5 | 12.0 | 33.0 |
| Distrito Federal | 14.5 | 33.6 | 10.3 | 26.7 | 12.5 | 27.6 | 12.4 | 29.3 |
| Espírito Santo | 12.7 | 32.3 | 9.7 | 27.6 | 11.5 | 27.8 | 11.3 | 29.2 |
| Goiás | 9.9 | 30.7 | 8.5 | 25.3 | 9.9 | 27.8 | 9.4 | 27.9 |
| Maranhão | 13.2 | 33.3 | 13.8 | 28.5 | 11.3 | 34.3 | 12.8 | 32.0 |
| Mato Grosso | 9.9 | 31.4 | 9.0 | 27.3 | 11.0 | 26.5 | 10.0 | 28.4 |
| Mato Grosso do Sul | 9.0 | 30.3 | 10.4 | 27.4 | 9.8 | 31.1 | 9.7 | 29.6 |
| Minas Gerais | 9.0 | 25.6 | 8.2 | 23.8 | 9.8 | 23.8 | 9.0 | 24.4 |
| Pará | 11.8 | 28.7 | 12.2 | 32.8 | 11.4 | 30.1 | 11.8 | 30.5 |
| Paraíba | 16.7 | 39.6 | 14.2 | 35.0 | 12.8 | 34.6 | 14.6 | 36.4 |
| Paraná | 9.9 | 24.7 | 10.2 | 26.0 | 8.7 | 25.9 | 9.6 | 25.5 |
| Pernambuco | 12.9 | 35.4 | 11.9 | 35.2 | 12.5 | 33.4 | 12.4 | 34.7 |
| Piauí | 12.9 | 31.2 | 13.0 | 33.5 | 12.6 | 33.3 | 12.8 | 32.7 |
| Rio de Janeiro | 8.5 | 28.1 | 9.7 | 31.4 | 9.2 | 31.8 | 9.1 | 30.4 |
| Rio Grande do Norte | 13.5 | 35.0 | 11.7 | 38.3 | 12.6 | 34.6 | 12.6 | 36.0 |
| Rio Grande do Sul | 11.4 | 28.3 | 8.6 | 27.5 | 8.4 | 26.3 | 9.5 | 27.4 |

| | | | | | | | | |
|---|---|---|---|---|---|---|---|---|
| Rondônia | 11.2 | 28.6 | 11.2 | 28.4 | 9.9 | 22.0 | 10.8 | 26.3 |
| Roraima | 11.1 | 29.2 | 10.2 | 30.7 | 10.3 | 28.4 | 10.5 | 29.4 |
| Santa Catarina | 10.5 | 34.4 | 8.9 | 29.6 | 10.3 | 31.4 | 9.9 | 31.8 |
| São Paulo | 11.1 | 28.9 | 9.6 | 28.8 | 6.4 | 24.3 | 9.0 | 27.3 |
| Sergipe | 11.3 | 32.2 | 14.0 | 29.6 | 11.3 | 26.0 | 12.2 | 29.3 |
| Tocantins | 10.5 | 31.3 | 11.5 | 27.9 | 9.4 | 27.6 | 10.5 | 28.9 |

In women aged 20-59 years (Fig 1A) and those aged ≥60 years (Fig 1B), respectively, 4.3% and 10.9% of hospitalizations by breast cancer were attributable to physical inactivity. The highest FAP values were found in Brazilian states of Northern, Northeastern and Midwestern regions, indicating that such locations would prevent greater number of hospitalizations by breast cancer if the population practiced physical activity regularly.

**Fig 1. Population Attributable Fraction indicating the percentage of hospitalizations for breast cancer due to physical inactivity in women aged 20-59 years (Fig 1A) and in women aged ≥ 60 years (Fig 1B) from the states of Brazil.**

The total hospitalization cost by breast cancer in women aged 20-59 years in Brazil from 2015 to 2017 was US$ 75,837,628.88. Of this total, US$ 3,224,043.20 was due to physical inactivity. The states of São Paulo (US$ 606,839.65) and Minas Gerais (US$ 327,765.01) were those with the highest hospitalization costs by breast cancer due to physical inactivity (Table 4). The total hospitalization cost by breast cancer in women ≥60 years of age in Brazil from 2015 to 2017 was US$

41,286,236.58. Of this total, US$ 4,260,623.66 was due to physical inactivity. The states of São Paulo (US$ 1,021,365.55) and Minas Gerais (US$ 394,464.87) were those with the highest hospitalization costs by breast cancer due to physical inactivity (Table 5).

**Table 4. Cost of hospitalizations for breast cancer in women aged 20 to 59 years in Brazil and in the states in 2015, 2016 and 2017.**

| | Total cost of hospitalizations by breast cancer (US$) | | | | Total cost of hospitalizations by breast cancer due to physical inactivity (US$) | | | |
|---|---|---|---|---|---|---|---|---|
| | **2015** | **2016** | **2017** | **Total** | **2015** | **2016** | **2017** | **Total** |
| Brazil | 23,645,812.45 | 25,158,500.14 | 27,033,316.30 | 75,837,628.88 | 1,048,514.62 | 1,060,319.93 | 1,109,537.72 | 3,224,043.20 |
| Acre | 10,776.12 | 14,631.41 | 21,276.50 | 46,684.03 | 513.15 | 578.96 | 865.43 | 1,957.54 |
| Alagoas | 229,939.20 | 261,786.36 | 413,522.40 | 905,247.96 | 10,280.05 | 12,466.02 | 22,225.66 | 44,971.72 |
| Amapá | 7,820.77 | 8,505.90 | 12,522.78 | 28,849.46 | 355.35 | 370.95 | 532.37 | 1,258.68 |
| Amazonas | 106,043.47 | 145,736.98 | 154,818.00 | 406,598.45 | 4,274.31 | 6,515.56 | 6,524.90 | 17,314.76 |
| Bahia | 2,412,804.00 | 2,558,231.52 | 2,406,249.06 | 7,377,284.58 | 99,916.73 | 101,228.52 | 77,320.43 | 278,465.68 |
| Ceará | 1,063,152.71 | 1,080,032.16 | 1,266,624.92 | 3,409,809.80 | 52,935.39 | 49,860.32 | 51,986.52 | 154,782.23 |
| Distrito Federal | 211,150.13 | 260,922.24 | 301,789.37 | 773,861.74 | 11,575.34 | 10,324.62 | 14,370.92 | 36,270.88 |
| Espírito Santo | 714,037.91 | 686,877.95 | 704,322.53 | 2,105,238.39 | 34,519.53 | 25,655.43 | 30,974.03 | 91,149.00 |
| Goiás | 516,042.67 | 743,625.77 | 794,167.63 | 2,053,836.07 | 19,656.88 | 24,451.91 | 30,251.09 | 74,359.88 |
| Maranhão | 444,793.79 | 468,503.72 | 565,600.04 | 1,478,897.56 | 22,307.29 | 24,508.53 | 24,459.55 | 71,275.37 |
| Mato Grosso | 209,470.52 | 263,415.98 | 295,959.00 | 768,845.51 | 7,979.06 | 9,153.45 | 12,473.37 | 29,605.88 |
| Mato Grosso do Sul | 236,205.85 | 281,170.12 | 305,558.85 | 822,934.82 | 8,207.93 | 11,229.53 | 11,526.08 | 30,963.54 |
| Minas Gerais | 2,911,826.89 | 3,052,656.33 | 3,436,639.12 | 9,401,122.34 | 101,183.17 | 96,947.26 | 129,634.58 | 327,765.01 |
| Pará | 244,281.50 | 253,405.20 | 347,934.34 | 845,621.04 | 11,010.40 | 11,790.78 | 15,173.88 | 37,975.06 |
| Paraíba | 407,817.15 | 374,064.13 | 487,374.31 | 1,269,255.59 | 25,536.36 | 20,104.89 | 23,738.17 | 69,379.41 |
| Paraná | 1,732,171.39 | 1,954,145.87 | 2,254,305.94 | 5,940,623.19 | 65,981.13 | 76,603.72 | 75,811.60 | 218,396.46 |

| | | | | | | | | |
|---|---|---|---|---|---|---|---|---|
| Pernambuco | 1,193,541.85 | 1,422,867.65 | 1,542,778.69 | 4,159,188.18 | 58,564.81 | 64,651.11 | 73,465.65 | 196,681.57 |
| Piauí | 303,727.69 | 380,054.91 | 430,461.00 | 1,114,243.60 | 14,903.34 | 18,785.98 | 20,654.26 | 54,343.58 |
| Rio de Janeiro | 1,563,482.54 | 1,577,146.01 | 1,669,392.41 | 4,810,020.96 | 51,410.45 | 58,907.65 | 59,253.13 | 169,571.23 |
| Rio Grande do Norte | 549,186.88 | 504,906.24 | 580,846.43 | 1,634,939.56 | 28,136.71 | 22,573.19 | 27,870.01 | 78,579.91 |
| Rio Grande do Sul | 1,602,418.38 | 1,509,308.11 | 1,682,746.42 | 4,794,472.92 | 69,883.59 | 50,193.54 | 54,702.28 | 174,779.41 |
| Rondônia | 116,165.61 | 159,478.34 | 173,108.21 | 448,752.16 | 4,981.07 | 6,838.27 | 6,593.96 | 18,413.31 |
| Roraima | 10,719.41 | 17,794.27 | 15,317.33 | 43,831.01 | 455.71 | 697.55 | 606.10 | 1,759.36 |
| Santa Catarina | 979,311.50 | 1,120,884.16 | 1,133,422.18 | 3,233,617.84 | 39,473.21 | 38,531.75 | 44,849.21 | 122,854.16 |
| São Paulo | 5,709,499.08 | 5,877,091.22 | 5,880,382.38 | 17,466,972.68 | 242,724.78 | 217,334.65 | 146,780.22 | 606,839.65 |
| Sergipe | 61,283.77 | 82,284.25 | 101,983.75 | 245,551.78 | 2,650.24 | 4,363.56 | 4,410.32 | 11,424.11 |
| Tocantins | 98,141.66 | 98,973.32 | 54,212.70 | 251,327.68 | 3,955.81 | 4,352.56 | 1,964.53 | 10,272.89 |

Note. US$: US American Dollar; US$ 1.00 = R$ 0.30 (R$ = Brazilian currency; mean of Brazilian currency from the values of 2015, 2016 and 2017).

**Table 5. Cost of hospitalizations for breast cancer in women aged ≥ 60 years in Brazil and in the states in 2015, 2016 and 2017.**

| | Total cost of hospitalizations by breast cancer (US$) | | | | Total cost of hospitalizations by breast cancer due to physical inactivity (US$) | | | |
|---|---|---|---|---|---|---|---|---|
| | 2015 | 2016 | 2017 | Total | 2015 | 2016 | 2017 | Total |
| Brazil | 13.078.807,34 | 13.508.413,48 | 14.699.015,76 | 41.286.236,58 | 1.391.073,22 | 1.400.794,55 | 1.468.755,89 | 4.260.623,66 |
| Acre | 2.111,06 | 4.034,17 | 4.734,52 | 10.879,74 | 278,54 | 474,19 | 566,81 | 1.319,54 |
| Alagoas | 116.948,89 | 142.089,59 | 189.492,16 | 448.530,64 | 14.934,85 | 18.188,66 | 22.744,39 | 55.867,91 |
| Amapá | 2.959,49 | 1.145,49 | 4.601,28 | 8.706,26 | 386,92 | 133,57 | 498,85 | 1.019,34 |
| Amazonas | 34.217,08 | 50.152,06 | 74.346,09 | 158.715,23 | 3.633,35 | 5.501,01 | 6.881,40 | 16.015,76 |
| Bahia | 1.033.459,62 | 964.092,19 | 910.467,31 | 2.908.019,13 | 126.279,48 | 106.664,06 | 101.306,89 | 334.250,42 |
| Ceará | 426.144,96 | 431.594,44 | 535.580,28 | 1.393.319,68 | 49.158,97 | 50.057,63 | 63.287,26 | 162.503,87 |
| Distrito Federal | 97.057,51 | 114.466,80 | 116.675,58 | 328.199,89 | 11.499,06 | 11.045,41 | 11.600,31 | 34.144,77 |
| Espírito Santo | 350.904,46 | 389.235,85 | 380.052,27 | 1.120.192,58 | 40.149,54 | 38.699,24 | 38.032,59 | 116.881,37 |
| Goiás | 249.770,78 | 315.604,47 | 324.780,69 | 890.155,94 | 27.317,29 | 29.003,97 | 32.501,45 | 88.822,71 |
| Maranhão | 182.838,17 | 198.900,89 | 256.158,37 | 637.897,42 | 21.491,39 | 20.354,31 | 30.904,79 | 72.750,49 |
| Mato Grosso | 107.486,50 | 100.845,14 | 119.109,15 | 327.440,80 | 11.993,87 | 9.928,14 | 11.415,52 | 33.337,54 |
| Mato Grosso do Sul | 144.210,30 | 155.935,17 | 159.687,03 | 459.832,50 | 15.588,91 | 15.402,39 | 17.667,26 | 48.658,56 |
| Minas Gerais | 1.716.677,45 | 1.845.389,50 | 2.136.234,30 | 5.698.301,25 | 159.459,15 | 160.410,04 | 185.691,66 | 505.560,86 |
| Pará | 114.905,40 | 108.890,60 | 144.807,45 | 368.603,46 | 11.832,74 | 12.629,46 | 15.561,24 | 40.023,45 |
| Paraíba | 208.267,69 | 222.119,83 | 233.537,99 | 663.925,51 | 28.478,59 | 27.277,87 | 28.392,18 | 84.148,65 |

| | | | | | | | | |
|---|---|---|---|---|---|---|---|---|
| Paraná | 954.761,79 | 1.090.210,58 | 1.226.641,95 | 3.271.614,31 | 85.848,62 | 102.701,00 | 115.150,51 | 303.700,13 |
| Pernambuco | 648.412,31 | 673.242,92 | 786.959,83 | 2.108.615,06 | 80.426,75 | 83.093,10 | 92.746,85 | 256.266,70 |
| Piauí | 166.010,64 | 218.276,24 | 204.102,91 | 588.389,79 | 18.419,39 | 25.792,78 | 23.990,92 | 68.203,09 |
| Rio de Janeiro | 1.173.073,73 | 1.226.769,50 | 1.232.168,13 | 3.632.011,36 | 118.530,64 | 136.888,99 | 139.045,23 | 394.464,87 |
| Rio Grande do Norte | 274.508,52 | 233.794,78 | 287.490,76 | 795.794,05 | 33.711,57 | 31.059,10 | 34.951,44 | 99.722,12 |
| Rio Grande do Sul | 1.094.119,36 | 1.045.962,92 | 1.106.892,24 | 3.246.974,52 | 111.259,71 | 103.653,98 | 105.361,08 | 320.274,77 |
| Rondônia | 59.113,02 | 51.546,61 | 55.046,54 | 165.706,18 | 6.068,31 | 5.258,35 | 4.452,29 | 15.778,96 |
| Roraima | 7.235,97 | 7.233,49 | 4.421,80 | 18.891,26 | 756,77 | 791,12 | 451,07 | 1.998,97 |
| Santa Catarina | 433.469,79 | 472.517,99 | 542.815,82 | 1.448.803,60 | 52.430,95 | 50.023,36 | 60.570,07 | 163.024,38 |
| São Paulo | 3.422.717,89 | 3.370.978,02 | 3.594.992,58 | 10.388.688,50 | 354.666,72 | 348.221,55 | 318.477,29 | 1.021.365,55 |
| Sergipe | 28.756,59 | 31.774,14 | 33.829,40 | 94.360,13 | 3.281,23 | 3.363,79 | 3.186,83 | 9.831,84 |
| Tocantins | 28.668,38 | 41.610,10 | 33.389,32 | 103.667,80 | 3.189,91 | 4.177,48 | 3.319,69 | 10.687,07 |

Note. US$: US American Dollar; US$ 1.00 = R$ 0.30 (R$ = Brazilian currency; mean of Brazilian currency from the values of 2015, 2016 and 2017).

The mean hospitalization cost for breast cancer due to physical inactivity in the period from 2015 to 2017 was higher in the Brazilian states of Southeastern and Southern regions (Fig 2). Both in women aged 20-59 years (rho = .37, p = .04) and in those aged ≥60 years (rho = .43, p = .02), the mean hospitalization cost due to physical inactivity was higher in Brazilian states with better levels of quality and access to health services (Fig 3).

**Fig 2. Average cost of hospitalizations for breast cancer due to physical inactivity in the period of 2015, 2016 and 2017 in the states of Brazil in women aged 20-59 years (Fig 2A) and in women aged ≥ 60 years (Fig 2B).** US$: US American Dollar; US$ 1.00 = R$ 0.30 (R$ = Brazilian currency; mean of Brazilian currency from the values of 2015, 2016 and 2017).

**Fig 3. Relationship between breast cancer hospitalization costs due to physical inactivity and the healthcare quality and Access index of Brazilian states in women aged 20-59 years (Fig 3A) and in women aged ≥ 60 years (Fig 3B).** Rho: Spearman's correlation coefficient.

# Discussion

The economic burden of physical inactivity was studied by authors from different countries who found high costs for health systems due to physical inactivity [29-31]. Most of these studies associated physical inactivity to costs due to cardiovascular disease or diabetes [29-31] and few estimated costs due to breast cancer [7]. The few studies that associated costs to breast cancer were focused on

leisure-time physical inactivity [7] and not with physical inactivity in all domains (leisure, transport, domestic activities, work), as was the case in the present study. The estimate of physical inactivity in all domains is important because depending on contextual and social characteristics, the population can be physically active in one domain and not in another [32]. In this sense, estimates of physical inactivity in only one domain may overestimate the economic burden of physical inactivity due to a given chronic disease.

The present study found that from 2015 to 2017, US$ 3,224,043.20 (annual mean value of US$ 1,072,790.76) were spent on hospitalizations for breast cancer due to physical inactivity in women aged 20-59 years and that US$ 4,260,623,66 (average annual value of US$ 1,420,207.89) were spent with women aged ≥60 years. These values reflected a total cost of more than seven million dollars in three years due to physical inactivity in Brazil. The values estimated in the present study are much higher than those used to create community programs to promote physical activity. Research carried out in Germany [33] investigated the costs for implementing physical activity programs in primary care and found that there are three phases for developing these programs: 1) evaluation of health assets; 2) development of preventive intervention programs involving political agents and society; 3) implementation of the program itself. The authors estimated different cost scenarios and reported that the highest estimated cost would be € 133,074, which is equivalent to approximately US$ 151,000,000 [33]. This value is equivalent to only 14% of the annual mean hospitalization cost of breast cancer due to physical inactivity from 20115 to 2017 in Brazil.

The federal government of Brazil developed the “Academia da Saúde” Program, which is a community program whose objective is to promote physical

activity, healthy eating, health education, contributing to the production of care and healthy and sustainable ways of life for the population. To this end, the Program promotes the implementation of public spaces with infrastructure, equipment and qualified professionals [34]. The practice of physical activity is one of the main actions of the program and the entire Brazilian population living in cities where the program has been implemented can participate free of charge [34]. The cost for the implementation of this program varies from R$ 80.000,00 (US$ 266,400.00) to R$ 180.000,00 (US$ 599,400.00) and has the capacity to serve the entire population of cities [34]. That is, the cost for cities to implement community-based programs to promote physical activity is lower than the hospitalization cost for breast cancer due to physical inactivity shown in the present study.

The development of programs to promote physical activity in workplaces can also be effective in increasing the levels of physical activity in the population, especially in the adult population that spends most of the day at work [35] and thus can be a strategy for governments to lower the costs of physical inactivity. Lutz et al. [35] developed a systematic review of programs aimed at promoting physical activity in the workplace and estimated the costs of these programs. Of the 16 studies that estimated the costs of implementing this type of program, an average cost per person of € 174.00 was reported, which is equivalent to approximately US$ 195.00. In other words, with estimated cost of US$ 195.00 per person in the work environment, physical activity promotion programs can be implemented, which will prevent breast cancer and other non-communicable diseases and conditions and will consequently reduce the economic burden of hospitalizations due to physical inactivity.

The present study found that in Brazilian women aged 20-59 years and those aged ≥60 years, respectively, 4.3% and 10.9% of hospitalizations for breast cancer could be avoided with the regular practice of physical activities. These results reinforce the beneficial effect of the practice of physical activity for the prevention of breast cancer in all age groups, mainly because the practice of physical activity causes changes in female sex hormones (estrogens and progesterone) and in body fat, factors that are related to higher risk of breast cancer [36].

The Brazilian states of Northern, Northeastern and Midwestern regions showed the highest FAP values, indicating that such locations would prevent greater number of hospitalizations by breast cancer if the population practiced physical activity on a regular basis. This information is useful in terms of public health because these geographical regions are those with the worst economic and social indicators, which also reflects in worse levels of quality and access to health services [3]. Thus, increasing the level of physical activity of the population in these geographic regions would have a beneficial effect in reducing hospitalizations by breast cancer, which would bring important economic results to these locations, also preventing women from being hospitalized and not having the adequate care and treatment for breast cancer, as these locations are those with poor infrastructure and fewer health professionals to serve the population compared to other Brazilian regions [3].

The Southern and Southeastern regions of Brazil have the best social, economic and quality indicators and access to health services [3]. These geographic regions were those that showed the highest hospitalization costs for breast cancer due to physical inactivity in the present study. Silva et al. [3] developed a survey with 26-year data (1990-2015) to estimate mortality by breast cancer due to physical

inactivity in Brazil and found that the most socially and economically developed Brazilian regions were those with higher standardized mortality rate for breast cancer due to physical inactivity over the 25 years of investigation. The authors argued that this occurs because these regions have the best health services and receive more people for the treatment of breast cancer, which could result in higher mortality rates [3]. The present study corroborates these results and shows higher hospitalization rates for breast cancer in the Southern and Southeastern regions of Brazil, which results in higher hospitalization costs. In addition, these more developed geographic regions have daily dynamic with greater exposure to risk factors for breast cancer, such as inadequate nutrition, higher prevalence of obesity and lower pregnancy rates compared to women living in other geographic regions [37].

As reported by other authors [7], the present study developed an analysis of the economic burden of physical inactivity from the direct cost related to expenses with a disease (i.e., hospitalization by breast cancer). This is because cost analysis can only be estimated from variables with measurable values and, therefore, can be accounted for, as is the case with hospitalization. However, regular physical activity can provide numerous health benefits that are considered immeasurable, such as its effects on mental well-being and improvements in quality of life [8]. Therefore, higher levels of physical activity in the population promote measurable and immeasurable effects on economy and health.

This study has several limitations, such as the estimation of hospitalization cost only for breast cancer. Physical inactivity is associated with other health problems that were not investigated in the present study [24], that is, the cost of physical inactivity for the health sector is significantly higher than that estimated in

this research. Thus, the results of this study on the economic burden of physical inactivity are much more worrying. Another limitation of this study is the non-stratification of hospitalizations by types of breast cancer and the non-exclusion of hospitalizations due to genetic mutations, as there are types of breast cancer that are more related to lifestyle than others [38]. This study developed analyses at level of geographic region and states of the country and not at level of municipality. The strategy used in the present study limits the identification of which municipalities have high hospitalization costs due to physical inactivity and which would need to invest more in physical activity programs in primary care. Finally, estimates of physical inactivity in the present study came from surveys that used self-reported physical activity measures, which are less accurate than objective measures [39].

# Conclusions

It could be concluded that physical inactivity has contributed to high number of hospitalizations by breast cancer in Brazilian adult and elderly women, which resulted in cost of more than seven million dollars from 2015 to 2017 for health services. In addition, the most socially and economically developed geographic regions with the best indicators of access to health services were those with the greatest economic burden of physical inactivity. On the other hand, the less socially and economically developed geographic regions of Brazil would have lower number of hospitalizations by breast cancer if the population meet the physical activity guidelines. Thus, the promotion of physical activity in the Brazilian female population would bring economic benefits and access to health services for all geographic regions.

# References

1. Kerr J, Anderson C, Lippman SM. Physical activity, sedentary behaviour, diet, and cancer: an update and emerging new evidence. Lancet Oncol. 2017;18(8):e457-71. doi: https://doi.org/10.1016/S1470-2045(17)30411-4
2. Global Burden of Disease Cancer Collaboration. Global, Regional, and National Cancer Incidence, Mortality, Years of Life Lost, Years Lived With Disability, and Disability-Adjusted Life-years for 32 Cancer Groups, 1990 to 2015: A Systematic Analysis for the Global Burden of Disease Study. JAMA Oncol. 2017;3(4):524-48. doi: https://doi.org/10.1001/jamaoncol.2016.5688
3. Silva DAS, Tremblay MS, Souza MFM, Guerra MR, Mooney M, Naghavi M, et al. Mortality and years of life lost due to breast cancer attributable to physical inactivity in the Brazilian female population (1990-2015). Sci Rep. 2018;8(1):11141. doi: https://doi.org/10.1038/s41598-018-29467-7
4. Heras BBL, Ramon Y Cajal JC, Calvo EG, Rodriguez JH, Mata JG, et al. The economic burden of metastatic breast cancer in Spain. Eur J Hosp Pharm. 2020;27(1):19-24. doi: https://doi.org/10.1136/ejhpharm-2017-001453
5. Unar-Munguía M, Meza R, Colchero MA, Torres-Mejía G, Cosío TG. Economic and disease burden of breast cancer associated with suboptimal breastfeeding practices in Mexico. Cancer Causes Control. 2017;28(12):1381-91. doi: https://doi.org/10.1007/s10552-017-0965-0
6. Sorensen SV, Goh JW, Pan F, Chen C, Yardley D, Martín M, et al. Incidence-based cost-of-illness model for metastatic breast cancer in the United States. Int J Technol Assess Health Care. 2012;28(1):12-21. doi: https://doi.org/10.1017/S026646231100064X

7. Bielemann RM, Silva BGC, Coll CVN, Xavier MO, Silva SG. Burden of physical inactivity and hospitalization costs due to chronic diseases. Rev Saúde Pública. 2015; 49:75. doi: https://doi.org/10.1590/S0034-8910.2015049005650
8. Bueno DR, Marucci Mde F, Codogno JS, Roediger MA. The Costs of Physical Inactivity in the World: A General Review. Cien Saude Colet. 2016;21(4):1001-10. doi: https://doi.org/10.1590/1413-81232015214.09082015
9. Ding D, Lawson KD, Kolbe-Alexander TL, Finkelstein EA, Katzmarzyk PT, van Mechelen W, et al. The economic burden of physical inactivity: a global analysis of major non-communicable diseases. Lancet. 2016;388(10051):1311-24. doi: https://doi.org/10.1016/S0140-6736(16)30383-X
10. Szwarcwald CL, Souza Júnior PRB, Marques AP, Almeida WS, Montilla DER. Inequalities in healthy life expectancy by Brazilian geographic regions: Findings from the National health survey, 2013. Int J Equity Health 2016;15:141.
11. Ishitani LH, Teixeira RA, Abreu DMX, Paixão LMMM, França EB. Quality of mortality statistics' information: garbage codes as causes of death in Belo Horizonte, 2011-2013. Rev Bras Epidemiol. 2017;20(Suppl 01):34-45. doi: https://doi.org/10.1590/1980-5497201700050004
12. Queiroz BL, Freire FHMA, Gonzaga MR, Lima EEC. Completeness of death-count coverage and adult mortality (45q15) for Brazilian states from 1980 to 2010. Rev Bras Epidemiol. 2017;20(Suppl 01):21-33. doi: https://doi.org/10.1590/1980-5497201700050003

13. World Health Organization (WHO). International Classification of Diseases (ICD). Available from: http://www.who.int/classifications/icd/en/
14. Brasil. Ministério da Saúde. Departamento de Informática do SUS (DATASUS), 2020. Available from: https://datasus.saude.gov.br/
15. Brasil. Ministério da Saúde. Saúde da pessoa idosa: prevenção e promoção à saúde integral, 2020. Available from: https://saude.gov.br/saude-de-a-z/saude-da-pessoa-idosa
16. Brasil. Banco Central do Brasil. Estatísticas sobre câmbio, 2020. Available from: https://www.bcb.gov.br/estatisticas/estatisticascambio
17. Brasil. Ministério da Saúde. Secretaria de Vigilância em Saúde. VIGITEL Brasil 2015. Vigilância de fatores de risco e proteção para doenças crônicas por inquérito telefônico. Brasília: Ministério da Saúde, 2016.
18. Brasil. Ministério da Saúde. Secretaria de Vigilância em Saúde. VIGITEL Brasil 2016. Vigilância de fatores de risco e proteção para doenças crônicas por inquérito telefônico. Brasília: Ministério da Saúde, 2017.
19. Brasil. Ministério da Saúde. Secretaria de Vigilância em Saúde. VIGITEL Brasil 2017. Vigilância de fatores de risco e proteção para doenças crônicas por inquérito telefônico. Brasília: Ministério da Saúde, 2018.
20. Monteiro CA, Florindo AA, Claro RM, Moura EC. Validity of indicators of physical activity and sedentariness obtained by telephone survey. Rev Saúde Pública 2008; 42(4): 575-81. doi: https://doi.org/10.1590/S0034-89102008000400001
21. Moreira AD, Claro RM, Felisbino-Mendes MS, Velasquez-Melendez G. Validity and reliability of a telephone survey of physical activity in Brazil. Rev

Bras Epidemiol. 2017; 20(1):136-146. doi: https://doi.org/10.1590/1980-5497201700010012.

22. World Health Organization. Global Recommendations on Physical Activity for Health. Geneva: World Health Organization; 2010.

23. Brasil. Instituto Brasileiro de Geografia e Estatística (IBGE)/Diretoria de Pesquisas. Coordenação de População e Indicadores Sociais. Gerência de Estudos e Análises da Dinâmica Demográfica. Projeção da população do Brasil e Unidades da Federação por sexo e idade para o período 2000-2030, 2020. Available from: http://tabnet.datasus.gov.br/cgi/deftohtm.exe?ibge/cnv/projpopuf.def

24. Kyu HH, Bachman VF, Alexander LT, Mumford JE, Afshin A, Estep K, et al. Physical activity and risk of breast cancer, colon cancer, diabetes, ischemic heart disease, and ischemic stroke events: systematic review and dose-response meta-analysis for the Global Burden of Disease Study 2013. BMJ. 2016;354:i3857. doi: https://doi.org/10.1136/bmj.i3857

25. GBD 2015 Healthcare Access and Quality Collaborators. Healthcare Access and Quality Index based on mortality from causes amenable to personal health care in 195 countries and territories, 1990–2015: a novel analysis from the Global Burden of Disease Study 2015. Lancet. 2017; 390(10091): 231-66. doi: https://doi.org/10.1016/S0140-6736(17)30818-8

26. GBD 2016 Healthcare Access and Quality Collaborators. Measuring Performance on the Healthcare Access and Quality Index for 195 Countries and Territories and Selected Subnational Locations: A Systematic Analysis From the Global Burden of Disease Study 2016. Lancet.

2018;391(10136):2236-71. doi: https://doi.org/10.1016/S0140-6736(18)30994-2

27. Lau H, Khosrawipour V, Kocbach P, Mikolajczyk A, Ichii H, Schubert J, et al. Internationally lost COVID-19 cases. J Microbiol Immunol Infect. 2020;53(3):454-8. doi: https://doi.org/10.1016/j.jmii.2020.03.013
28. GBD 2017 Oesophageal Cancer Collaborators. The global, regional, and national burden of oesophageal cancer and its attributable risk factors in 195 countries and territories, 1990-2017: a systematic analysis for the Global Burden of Disease Study 2017. Lancet Gastroenterol Hepatol. 2020;5(6):582-97. doi: https://doi.org/10.1016/S2468-1253(20)30007-8
29. Huang CJ, Lin CH, Hsieh HM, Chang CC, Chu CC, Sun DP, et al. A longitudinal study of healthcare utilisation and expenditure in people with type 2 diabetes mellitus with and without major depressive disorder. Gen Hosp Psychiatry. 2019;57:50-8. doi: https://doi.org/10.1016/j.genhosppsych.2018.09.007
30. Su CL, Wang L, Ho CC, Nfor ON, Hsu S, Lee C, et al. Physical activity is associated with lower health care costs among Taiwanese individuals with diabetes mellitus. Medicine (Baltimore). 2020;99(14):e19613. doi: https://doi.org/10.1097/MD.0000000000019613
31. Crosland P, Ananthapavan J, Davison J, Lambert M, Carter R. The economic cost of preventable disease in Australia: a systematic review of estimates and methods. Aust N Z J Public Health. 2019;43(5):484-95. doi: https://doi.org/10.1111/1753-6405.12925
32. Paudel S, Owen AJ, Heritier S, Smith BJ. Prevalence of Total and Domain-Specific Physical Activity and Associated Factors Among Nepalese Adults: A

Quantile Regression Analysis. J Phys Act Health. 2020;17(5):501-11. doi: https://doi.org/10.1123/jpah.2019-0441

33. Wolfenstetter SB, Schweikert B, John J. Programme costing of a physical activity programme in primary prevention: should the costs of health asset assessment and participatory programme development count? Adv Prev Med. 2012;2012:601631. doi: https://doi.org/10.1155/2012/601631

34. Sá GB, Dornelles GC, Cruz KG, Amorim RC, Andrade SS, Oliveira TP, et al. The Health Academy Program as a strategy to promote health and healthy lifestyles: the national implementation scenario. Cien Saude Colet. 2016;21(6):1849-60. doi: https://doi.org/10.1590/1413-81232015216.09562016

35. Lutz N, Clarys P, Koenig I, Deliens T, Taeymans J, Verhaeghe N. Health economic evaluations of interventions to increase physical activity and decrease sedentary behavior at the workplace: a systematic review. Scand J Work Environ Health. 2020;46(2):127-42. doi: https://doi.org/10.5271/sjweh.3871

36. Colditz GA, Bohlke K. Priorities for the primary prevention of breast cancer. CA Cancer J Clin. 2014;64(3):186-194. doi: https://doi.org/10.3322/caac.21225

37. Adams, P. The breast cancer conundrum. Bull. World Health Organ. 91, 626–627 (2013).

38. Solà M, Recaj M, Castellà E, Puig P, Gubern JM, Julian JF, Fraile M. Sentinel Node Biopsy in Special Histologic Types of Invasive Breast Cancer. J Breast Health. 2016;12(2):78-82. doi: https://doi.org/10.5152/tjbh.2016.2929

39. Strath SJ, Kaminsky LA, Ainsworth BE, Ekelund U, Freedson PS, Gary RA, et al. Guide to the assessment of physical activity: Clinical and research applications: a scientific statement from the American Heart Association. Circulation. 2013;128(20):2259-79. doi: https://doi.org/10.1161/01.cir.0000435708.67487

# Supporting information

**S1 Table. Incidence of breast cancer in women in Brazil (≥ 20 years).** *Age-standardized (per 100,000 inhabitants).

Figure_1

Click here to access/download;Figure;Figure 1-.png

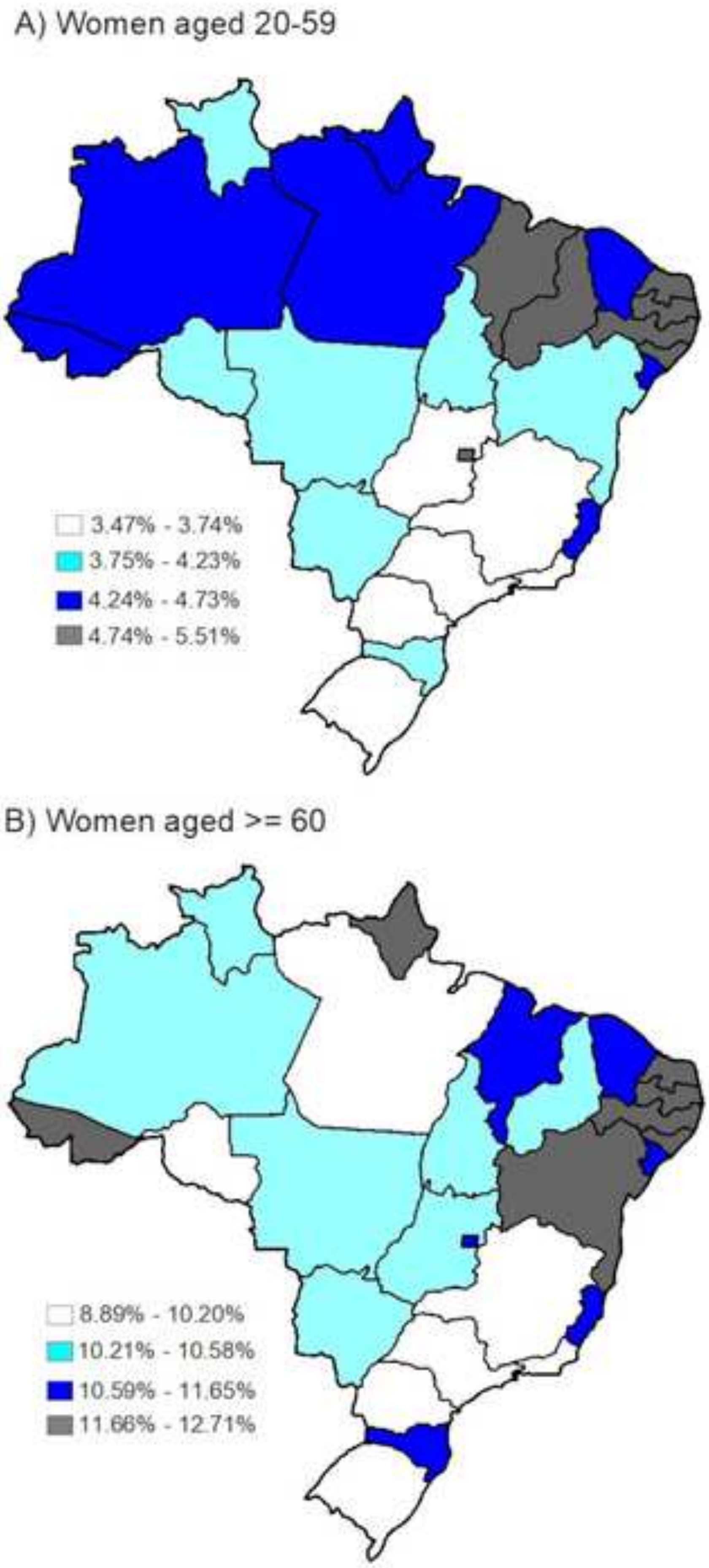

Figure_2

Click here to access/download;Figure;Figure 2-.png

A) Women aged 20-59

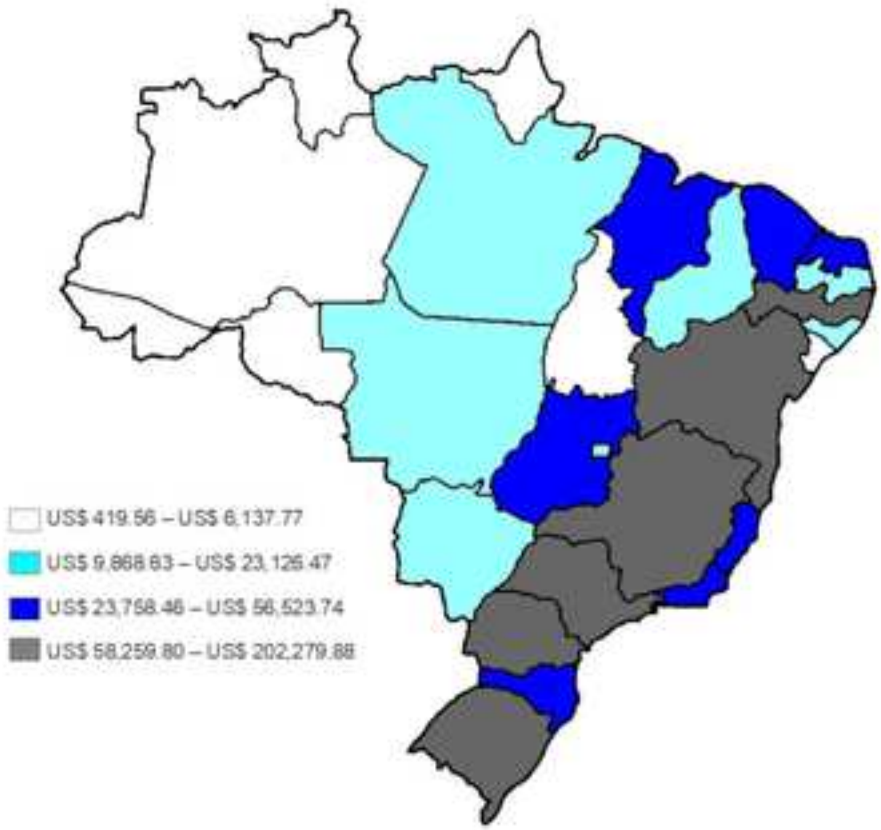


B) Women aged >= 60

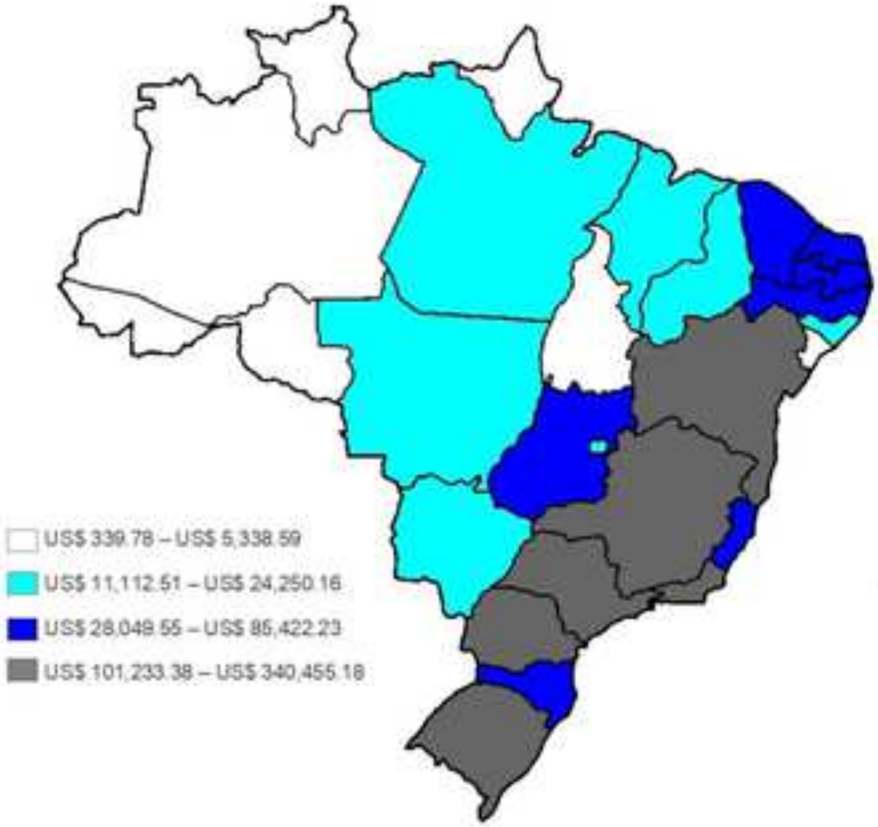

Figure_3

Click here to access/download;Figure;Figure 3-.png

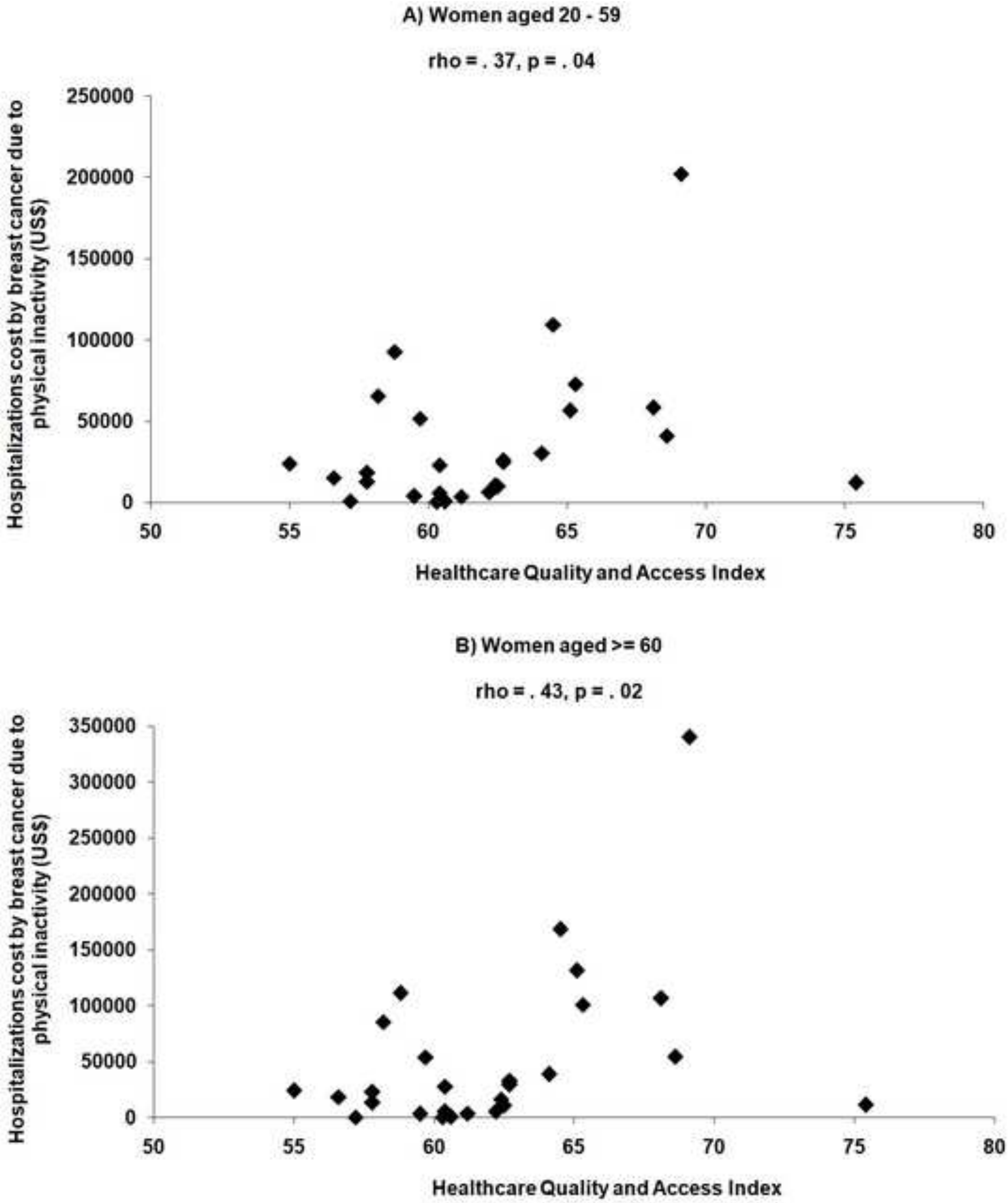

S1_Table

Click here to access/download
**Supporting Information**
Supplementary Table 1.pdf
