# Supplementary material for: Hospitalization and ambulatory costs related to breast cancer due to physical inactivity in the Brazilian state capitals
Source: PLoS One. 2022 Jan 19;17(1):e0261019. doi: 10.1371/journal.pone.0261019 (PMC8769291; doi:10.1371/journal.pone.0261019)
Supplement: Attachment — Submitted filename: Response to Reviewers.doc [file pone.0261019.s005.doc]

Florianopolis, Brazil, June 18, 2021.

**Muhammad Shahzad Aslam, Ph.D.,M.Phil., Pharm-D**

**Academic Editor - PLOS ONE**

**Dear Editorial Board of the PLOS ONE**,

Thank you for the careful review of our manuscript. We truly believe that all comments and suggestions have contributed to improve the paper. On behalf of all authors, I am submitting a revision of the manuscript entitled “Hospitalization cost related to breast cancer due to physical inactivity in the Brazilian state capitals” - No.: PONE-D-21-09895 - to your attention and for further review and consideration for publication in **PLOS ONE**. In addition, please find below details on the changes made to the original manuscript. The modifications made in the manuscript are marked in yellow.

Research has shown that there The total hospitalization cost by breast cancer in women aged 20-59 years in Brazil from 2015 to 2017 was US$ 21,230,637.04. Of this total, US$ 65,477.63 was due to physical inactivity. The total hospitalization cost by breast cancer in women ≥60 years of age in Brazil from 2015 to 2017 was US$ 3,678,860.25. Of this total, US$ 32,323.75 was due to physical inactivity. Both in Lower values of Healthcare Quality and Access index of the cities (for women aged 20-59 years: rho = – 0.62, p < 0.01; for women aged ≥60 years: rho = – 0.60, p < 0.01), was associated with higher percentage of hospitalizations by breast cancer due to physical inactivity. In women aged 20-59 years and those aged ≥60 years, respectively, 0.33% and 0.91% (PAF values) of hospitalizations by breast cancer were attributable to physical inactivity. Physical inactivity has contributed to the high number of hospitalizations for breast cancer in Brazil, which resulted in economic burden for health services of more than US$ 170,000 from 2015 to 2017.

We believe that the findings of this study are relevant to the scope of your journal and will be of interest to its readership. We have approved the manuscript and agree with submission to **PLOS ONE**. There are no conflicts of interest to declare.

The paper has not been published before, and it is not being considered for publication elsewhere and we approved the contents of the submitted manuscript. We declare that all authors have participated in the project design and / or analysis of the data obtained and / or the final writing of the article and that all agree with the version sent to the publication. In addition, no information has been omitted regarding research funding or liaison with individuals or companies that may be interested in the data addressed by the article. Authors are responsible for the content of the manuscript. We agree to assign exclusive copyright to the **PLOS ONE** if and when the manuscript is accepted for publication.

Best regards,

*Prof. Dr. Diego Augusto Santos Silva*

*Research Center in Kinanthropometry and Human Performance. Federal University of Santa Catarina. Florianopolis, Brazil. (E-mail: diegoaugustoss@yahoo.com.br)*

**Reviewer #1:**

The manuscript "The economic burden of breast cancer due to physical inactivity in Brazil" study the economic impact of physical inactivity and breast cancer to Brazilian healthcare system. The study has merit and was written. However, I have some comments to the author.

**Authors:** We really appreciate your comments on the article.

**Reviewer #1:**

Major comments:

1) There is only one author in the study? If is true, the sentence “These authors contributed equally to this work” in the first page make no sense.

**Authors:** The article has only one author. In the new version of the article the sentence was corrected to "This author contributed to all the work”.

**Reviewer #1:**

2) The correlation between mean hospitalization cost and healthcare quality and access index is weak both in women aged 20-59 years and in those aged ≥60 years (rho = 0.37, p = 0.04 and rho = 0.43, p = 0.02) (figure 1 and 2). Besides some Brazilian estates showed a higher cost and higher access index, this is not direct correlated in the country. This should be clear in results and discussion.

**Authors:** We really appreciate your comments. In the new version of the article we only added information about Brazilian capitals as suggested by other reviewers. These new data demonstrated that there was no relationship between mean hospitalization cost and healthcare quality and access index. In addition, in discussion section we added the information: “The Brazilian states of Northern and Northeastern regions showed the highest PAF values, indicating that such locations would prevent greater number of hospitalizations by breast cancer if the population practiced physical activity on a regular basis. In addition, the present study found that Brazilian capitals with lower values of Healthcare Quality and Access index had a higher percentage of hospitalizations by breast cancer due to physical inactivity. This information is useful in terms of public health because these geographical regions are those with the worst economic and social indicators, which also reflects in worse levels of quality and access to health services [3]. Thus, increasing the level of physical activity of the population in these geographic regions would have a beneficial effect in reducing hospitalizations by breast cancer, which would bring important economic results to these locations, also preventing women from being hospitalized and not having the adequate care and treatment for breast cancer, as these locations are those with poor infrastructure and fewer health professionals to serve the population compared to other Brazilian regions [3]”.

**Reviewer #1:**

3) In the discussion, lines 353 to 355 “The cost for the implementation of this program varies from R$ 80.000,00 (US$ 266,400.00) to R$ 180.000,00 (US$ 599,400.00) and ….”. The values in Real and Dollar should be revised in this sentence and perhaps in all text.

**Authors:** The change was made: “…The cost for the implementation of this program varies from R$ 80,000.00 (US$ 15,822.49) to R$ 180.000,00 (US$ 35,600.60)…”.

**Reviewer #1:**

Minor comments:

1) Values of rho and p should be corrected. For instance: rho = .37, p = .04 to rho= 0.37, p = 0.04

**Authors:** The change was made.

**Reviewer #2:** I found that the article is well written. The content is interesting and well elaborated.

I found same minor errors that should be seen.

**Authors:** We really appreciate your comments on the article.

**Reviewer #2:**

Line 62 probably the number in euros is wrongly written.

**Authors:** The change was made

**Reviewer #2:**

Line 185 the term PAF was used in line 251 the term FAP was used for the same meaning

**Authors:** In the new version of the paper we put all terms as “PAF”.

**Reviewer #2:**

Line 342 is this phrase correct?

would be € 133,074, which is equivalent to approximately US$ 151,000,000

The authors estimated different cost scenarios and reported that the highest estimated cost would be € 133,074.00, which is equivalent to approximately **Authors:** In the new version of the paper we correct the information: “…The authors estimated different cost scenarios and reported that the highest estimated cost would be € 133,074.00, which is equivalent to approximately US$ 162,040.00 [33] (€ 1.00 = US$ 1.22 in June 10, 2021)…”.

**Reviewer #2:**

Line 354

implementation of this program varies from R$ 80.000,00 (US$ 266,400.00) to R$ 354 180.000,00 (US$ 599,400.00)

**Authors:** In the new version of the paper we correct the information: “…The cost for the implementation of this program varies from R$ 80,000.00 (US$ 15,822.49) to R$ 180.000,00 (US$ 35,600.60) and has the capacity to serve the entire population of cities…”.

**Reviewer #3:**

The study is important to added the information about the correlation about a breast cancer and economic costs in populous country such as brazil. The Datasus is a main source of data health from Brazilian`s population. This study have not study design, and the authors did not declare the conflict of interest.

Please see attachment.

**Authors:** We really appreciate your comments on the article.

**Reviewer #4:**

General comments: The article has an exciting and very current theme. Economic analyzes in low- and middle-income countries are always welcome. Three methodological issues are central. The first question refers to the option of the authors to use a survey of the prevalence of risk factors that is not nationally representative to build a fraction attributable to be applied nationally. The second question concerns using only direct hospital expenses and highlighting in the title that an analysis of the economic burden is a broad concept. The third and last concerns the questionable contribution of the correlation analysis between attributable expenditure and the Healthcare Access and Quality (HAQ) Index.

**Authors:** We really appreciate your comments on the article. We agree that it is a limitation of the article to investigate only Brazilian capitals. This information was added in the discussion section as a limitation: “…This study developed analyses at level of Brazilian states capitals, and not at level of states. The strategy used in the present study limits the identification of which states or other cities of the Brazil have high hospitalization costs due to physical inactivity and which would need to invest more in physical activity programs in primary care...”.

In the new version of the paper we changed the title and the aim of this study.

Title: Hospitalization cost related to breast cancer due to physical inactivity in the Brazilian state capitals; Objective: to estimate the hospitalization cost related to breast cancer due to physical inactivity in the Brazilian states capitals over a three-year period (2015 to 2017) and to relate these costs to conditions of access and quality of health services in Brazilian states.

**Reviewer #4:**

Specific comments: 1. Although the article's title refers to economic burden in Brazil, data from Vigitel were used, which is a telephone survey with representation only for the 26 capitals and DF and not for Brazil;

**Authors:** We really appreciate your comments on the article. We agree that it is a limitation of the article to investigate only Brazilian capitals. Because of that we changed all the results. In addition, this information was added in the discussion section as a limitation: “…This study developed analyses at level of Brazilian states capitals, and not at level of states. The strategy used in the present study limits the identification of which states or other cities of the Brazil have high hospitalization costs due to physical inactivity and which would need to invest more in physical activity programs in primary care...”.

**Reviewer #4:**

2. The economic burden seems a broad term. The author only analyzed the hospital expenses of the public health system;

**Authors:** In the new version of the paper we changed the title and the aim of this study.

Title: Hospitalization cost related to breast cancer due to physical inactivity in the Brazilian state capitals; Objective: to estimate the hospitalization cost related to breast cancer due to physical inactivity in the Brazilian states capitals over a three-year period (2015 to 2017) and to relate these costs to conditions of access and quality of health services in Brazilian states.

**Reviewer #4:**

3. It was unclear why choosing the analysis period between 2015 and 2017;

**Authors:** We chose these years because they are the years with data consolidated in DATASUS. The most recent data available in DATASUS (2018, 2019 and 2020) are still in the process of consolidation by the national system.

**Reviewer #4:**

4. It was also unclear why analyzing the correlation between expenditures and conditions of access and quality of services (HAQ) in the states;

**Authors:** In the new version of the article we added new correlations between percentage of hospitalizations for breast cancer due to physical inactivity and the Healthcare Quality and Access index. The information allowed progress in the discussions of the article.

**Reviewer #4:**

4. It seems that the approval of CONEP was from the telephone survey research (Vigitel) and not from the study carried out;

**Authors:** The data collected directly from people was from VIGITEL, because of that the Ethics Committee was referring to this research. DATASUS data are secondary and free to access.

**Reviewer #4:**

5.What is the purpose of calculating the standardized rate of hospitalization for breast cancer;

**Authors:** It is important to calculate the standardized rate because the standardized rate takes into account the population of the city. If the rate is not standardized, the information will not be real for the population of interest (Please visit:

https://www.cdc.gov/cancer/uscs/technical_notes/stat_methods/rates.htm#:~:text=Crude%20rates%20are%20influenced%20by,cancers%20increase%20with%20increasing%20age).

**Reviewer #4:**

6. the value of RR 1.40 used was not found in the reference text;

**Authors:** In the new version of the paper we changed the RR. RR of the relationship between physical activity and breast cancer was obtained from meta-analysis that analyzed the dose-response between these variables with the inclusion of adjustment covariates based on studies with good methodological quality (Kyu et al, 2016). Kyu et al. (2016) reported that women who practiced physical activity in an amount ≥ 600 MET minutes/week of total physical activity had at least a reduced risk of breast cancer by 3% compared to those who reported lower amounts of physical activity. In the present study RR considered was 1.03, which indicated the risk of breast cancer in women who did not meet physical activity recommendations of 600 METs minutes/week.

**Reviewer #4:**

7. The example used in the discussion (lines 336-344) does not seem suitable for the discussion section once it was a foreign factual experience;

**Authors:** In the new version of the paper we removed the Germany article from the discussion.

**Reviewer #4:**

8. Review the dollar amounts in lines 352-355;

**Authors:** The change was made.

**Reviewer #4:**

9. The reference used in lines 358-370 also does not seem adequate since an analysis of the attributable fraction of expenses by type (recreational, occupational, transport, at home) was not performed. The values marked in the article were not found in the original

**Authors:** In the new version of the paper we kept the article in the discussion section because it brings a debate about the implementation of physical activity programs in other contexts, which can be a strategy to be used in Brazil.

The value marked in our research is in the pag. 135 of the paper of Lutz et al. (2020).

- Lutz N, Clarys P, Koenig I, Deliens T, Taeymans J, Verhaeghe N. Health economic evaluations of interventions to increase physical activity and decrease sedentary behavior at the workplace: a systematic review. Scand J Work Environ Health. 2020;46(2):127-42. doi: https://doi.org/10.5271/sjweh.3871

**Reviewer #4:**

10. Clarify the justification pointed out in lines 408-412; 11. The quality of life and mental well-being are considered non-measurable; why? Lines 412-416;

**Authors:** We really appreciate your comments on the article. In the new version of the paper we changed this paragraph: “...The present study developed an analysis of the hospitalization cost by breast cancer. This analysis is one of the possible analyzes for estimating the economic burden of a disease [7]. The cost analysis can only be estimated from variables with measurable values and, therefore, can be accounted for, as is the case with hospitalization. However, regular physical activity can provide numerous health benefits, such as its effects on the reduction in the use of medications [38], and in recovering from some illnesses [39]. Therefore, higher levels of physical activity in the population promote positive effects on economy and health…”.

**Reviewer #4:**

12. In the study's limitations, there was no mention of the issue of temporal GAP between exposure to the risk factor and the outcome. This question has not been considered, although other research groups on attributable fraction consider it very relevant.

**Authors:** In the new version of the paper we added this limitation.

**Reviewer #4:**

13. I could not access reference #38, review doi

**Authors:** The change was made.

**Reviewer #4:**

14. Lines 425-426 reinforce that attributable expenditures were carried out using prevalence data from the state's capitals. Wouldn't that be a limitation?

**Authors:** In the new version of the paper all the information was from state’s capitals.

**Reviewer #4:**

15. No analysis was made of the correlation between expenditure attributable to physical inactivity and the HAQ index. Why? since the rho values are relatively low.

**Authors:** In the new version of the article we added new correlations between percentage of hospitalizations for breast cancer due to physical inactivity and the Healthcare Quality and Access index. The information allowed progress in the discussions of the article.

**Reviewer #5:**

The introduction section is so long. Along the stretch between 62 and 81 lines are highlighted some studies with cost estimates widely different, limiting the cross-countries comparison. Sugiro excluir a maior parte e/ou transferir uma parte para a discussão. I suggest deleting most of this and/or transferring a part to the discussion.

**Authors:** The different reviewers of the paper have debated the different sections of the research. We have chosen to keep the introduction information because, along with other reviewers, we understand that it is important for the reader to bring information from previous studies about the costs of physical inactivity.

**Reviewer #5:**

It is not clear which is the study design.

**Authors:** Our paper is an ecological study. We added the information on the method section.

**Reviewer #5:**

The authors argue that “For all Brazilian states, the quality of data from DATASUS is considered high and close to high-income countries”. However, several authors suggest which coverage and quality of the North Brazilian region datasets is poor in regard south region, for example (Please, check this manuscripts: https://pubmed.ncbi.nlm.nih.gov/31800858/; <https://pubmed.ncbi.nlm.nih.gov/31859881/>).

**Authors:** The quality of data from DATASUS is considered high and close to high-income countries. I understand that some information still needs further improvement and it does need to improve. However, DATASUS is a quality system whose data is important for Brazil.

**Reviewer #5:**

Please consider exclude the following stretch “All DATASUS information follows the ethical precepts of the Declaration of Helsinki, so that users' anonymity is respected.”, because the study is based in secondary data.

**Authors:** The change was made.

**Reviewer #5:**

How the authors dealt with the number of readmissions?

**Authors:** We do not consider this information in the article.

**Reviewer #5:**

If the quality of data from DATASUS is considered high in Brazil why the authors did not use the breast cancer Brazilian estimates?

**Authors:** In the new version of the paper we added all the information from DATASUS (included breast cancer estimates). “…Information regarding the incidence of breast cancer in the Brazilian female population aged 20-59 years and aged ≥ 60 years over 2015, 2016 and 2017 was taken from estimates of the DATASUS (i.e., Panel-Oncology), which analyzes estimates and makes information available free of charge”.

**Reviewer #5:**

The Healthcare Access and Quality (HAQ) Index may have been influenced by the poor-quality data of the north region cities. Are the north region estimates reliable?

**Authors:** The quality of data from DATASUS is considered high and close to high-income countries. I understand that some information still needs further improvement and it does need to improve. However, DATASUS is a quality system whose data is important for Brazil.

**Reviewer #6:**

This study highlights an important topic on the impact of physical inactivity on hospitalization costs due to breast cancer, through secondary analysis of national databases. The study aimed “to estimate the economic burden of breast cancer due to physical inactivity in the Brazilian female population over a three-year period (2015 to 2017) and to relate these costs to conditions of access and quality of health services in Brazilian states”.

I have several major and some minor observations.

1) A first concern is using the VIGITEL database to estimate the prevalence of physical inactivity of Brazilians. This database is restricted to the Brazilian population aged ≥ 18 years living in households served by at least one fixed telephone line in 26 Brazilian capitals and the Federal District. Then, it is not representative of the Brazilian population, primarily because the exposure investigated (physical activity) can differ significantly between female residents of capitals and other cities in the country. The title, abstract, analyses, results, and conclusion should be revised to attend to this particularity.

**Authors:** We really appreciate your comments. In the new version of the article we only added information about Brazilian capitals as suggested.

**Reviewer #6:**

2) In the Abstract, the author stated that the “Population Attributable Fraction” (PAF) was calculated but did not present the results, an important result of this study.

**Authors:** The change was made.

**Reviewer #6:**

3) In the Introduction (lines 61-67), it stated some cost estimates of breast cancer in different countries and, importantly, in different points of time (from 2007 to 2020). This difference in time could be influencing in the estimates presented. I suggest considering presenting more current data and standardize the values in dollars.

**Authors:** The change was made for all information.

**Reviewer #6:**

4) In the Methods (lines 97-112), the databases used in the present study should be presented more clearly. Which data were obtained from DATAUS (SIH?) and which data were obtained from VIGITEL surveys? Are DATASUS and VIGITEL different databases with specific aims?

**Authors:** We used the information of the SIH for hospitalization due to breast cancer. The information about physical activity were from Vigitel. In the new version of the paper we added the information.

**Reviewer #6:**

5) In lines 99-100, the author stated that “the quality of data from DATASUS is considered high and close to high-income countries”, but the references used to support this claim (11,12) deal whit mortality statistics' information, not included in this study. Please review this claim and references.

**Authors:** The mortality statistics' information are from DATASUS. Because of that we kept the reference.

**Reviewer #6:**

6) In lines 129-132, it is stated the data extracted from the DATASUS (total number of hospitalizations and the cost of these hospitalizations in the Brazilian female population aged 20-59 years and ≥60 years, according to year). In lines 325-326, the author informs that analyses were conducted at the country's geographic region and states and not at municipality level. It is unclear to me why the analyses were not restricted to 26 Brazilian capitals and the Federal District?

**Authors:** We really appreciate your comments. In the new version of the article we only added information about Brazilian capitals. We changed all the results. In the new version of the article we added new correlations between percentage of hospitalizations for breast cancer due to physical inactivity and the Healthcare Quality and Access index. The information allowed progress in the discussions of the article.

**Reviewer #6:**

7) The RR used to estimate the attributable fraction was taken from a systematic review (Kyu 2013) (lines 192-195) that led to studies conducted in developed countries with populations different from the Brazilian one (United States, Canada, European countries, Japan and China). This limitation should be pointed out in the discussion.

**Authors:** We really appreciate your comments. We added this limitation.

**Reviewer #6:**

8) Please consider presenting the results by capitals and Federal District, not by Brazilian states.

**Authors:** In the new version of the article we only added information about Brazilian capitals. We changed all the results. In the new version of the article we added new correlations between percentage of hospitalizations for breast cancer due to physical inactivity and the Healthcare Quality and Access index. The information allowed progress in the discussions of the article.

**Reviewer #6:**

9) Lines 299-300. The statistic used (Spearman's correlation coefficient) was not described in the Method.

**Authors:** In the new version of the article we added the information.

**Reviewer #6:**

10) In lines 320-321, it is stated that “few estimated costs due to breast cancer [7]”. Please consider to cite other studies, such as Ding, 2016 (ref 9).

**Authors:** The changed was made.

**Reviewer #6:**

11) In lines 366-370, the author stated that “[…] physical activity promotion programs can be implemented, which will prevent breast cancer and other non-communicable diseases and conditions.” Despite the recognized importance of these programs, they do not guarantee the prevention of all breast cancer cases, given the existence of other risk factors for the disease.

**Authors:** In the new version of the article we added others information.

**Reviewer #6:**

12) It would be great if the author could include a limitation about the susceptibility of the analysis conducted in the present study to ecological fallacy.

**Authors:** In the new version of the article we added this limitation.

**Reviewer #6:**

13) Please change FAP (lines 251 and 379) by PAF.

**Authors:** The changed was made.
