# Supplementary material for: Hospitalization and ambulatory costs related to breast cancer due to physical inactivity in the Brazilian state capitals
Source: PLoS One. 2022 Jan 19;17(1):e0261019. doi: 10.1371/journal.pone.0261019 (PMC8769291; doi:10.1371/journal.pone.0261019)
Supplement: Attachment — Submitted filename: Response to Reviewers 3.doc [file pone.0261019.s006.doc]

**Reviewer #4:**

Thanks to the author for his availability to answer the questions asked by the reviewers. As for the manuscript, essential questions remain that still need to be clarified.

**Authors:** We really appreciate your comments. In the new version of the article we have added most of your suggestions. In other suggestions, we provide explanations based on the literature to exemplify our decisions.

**Reviewer #4:**

1. When re-reading the manuscript, after adjustments, it is not clear to the reader the reason for analyzing only hospital expenses and only expenses in capitals and DF. For most cancers, including breast cancer, ambulatory (outpatient) costs are much higher than hospital (inpatient) costs. Why not analyze both expenses? Why analyze only spending in capitals and DF? Wouldn't the expenses in the UF be more significant than in the capitals? Was the choice made for greater ease, convenience, or the availability in data collection, or is there any other reason?

**Authors:** We really appreciate your comments. In the new version of the article we added outpatient costs. Thus, this article presents hospitalization costs and outpatient costs.

The decision to analyze the capitals of Brazil and the Federal District was made because the survey of physical activity that is carried out in Brazil annually (e.g., VIGITEL) is carried out in Brazilian capitals and in the Federal District. Therefore, making estimates for all municipalities in Brazil based on physical activity information from Brazilian capitals would not be appropriate. We emphasize that there are no annual physical activity surveys covering all Brazilian states. These surveys only cover the capitals. In the new version of the paper we added this limitation: “This study developed analyses at level of Brazilian capitals, and not at level of states. The strategy used in the present study limits the identification of which states or other cities of the Brazil have high hospitalization and ambulatory costs due to physical inactivity and which would need to invest more in physical activity programs in primary care. The decision to analyze the capitals of Brazil and the Federal District was made because the survey of physical activity that is carried out in Brazil annually is carried out in Brazilian capitals and in the Federal District [17-19]”.

**Reviewer #4:**

2. The usefulness of presenting breast cancer incidence (number and raw rate) and hospitalization (number and age-standardized rate) data are not convincing. Where was this data used in the manuscript? What is the need for four tables to present unused data?

**Authors:** We really appreciate your comments. In the new version of the article we removed this information from the article. This information was presented as supplementary documents, as if readers want to know general information about breast cancer in Brazil, they can consult the supplementary files.

**Reviewer #4:**

3. It is unclear what is the usefulness of using the HAQ Index. There is no mention of a theoretical or empirical association between this indicator and the prevalence of physical inactivity in the introduction. What premise or assumption motivated the author to include this analysis?

**Authors:** We agree with the reviewer. In the new version of the article we removed the information about HAQ Index. This information really was without theoretical support.

**Reviewer #4:**

4. In the reference article by Kyu et al. (2016), the RR varies according to activity level. Compared with less active women (reporting less than 600 MET minutes/week of total physical activity), the risk of breast cancer among those in the low active (600-3999 MET minutes), moderately active (4000-7999 MET minutes), and highly active (≥8000 MET minutes) categories were, respectively, reduced by 3%, 6%, and 14%. The author used Vigitel's physically inactive women as a category of prevalence analysis. This category corresponds to how many METs/minutes/week? The impression is that the correct category of choice for Vigitel would be "insufficient physical activity" and not "physically inactive." Clarify this question, please.

**Authors:** We really appreciate your comments. In the new version of the article we added this information in the method section: “... Physically inactive were subjects who did not practice any free-time physical activity in the last three months of the interview and those who did not make intense physical efforts at work, did not commute to work or school and were not responsible for heavy cleaning of the house, as recommended in literature (e.g., they are those who have energy expenditure < 600 METS minutes/week) [22]”.

In 2017, a consensus was released (Tremblay et al., 2017) in which the concepts related to physical activity and sedentary behavior were defined. In this consensus was defined that physical inactivity is an insufficient physical activity level to meet present physical activity recommendations (i.e., < 600 METS minutes/week). For that reason, we used the term physical inactivity in our paper.

Reference:

Tremblay MS, Aubert S, Barnes JD, Saunders TJ, Carson V, Latimer-Cheung AE, Chastin SFM, Altenburg TM, Chinapaw MJM; SBRN Terminology Consensus Project Participants. Sedentary Behavior Research Network (SBRN) - Terminology Consensus Project process and outcome. Int J Behav Nutr Phys Act. 2017 Jun 10;14(1):75. doi: 10.1186/s12966-017-0525-8.

**Reviewer #4:**

5. According to the 2018 WCRF report on physical activity and cancer risk, physical activity has been associated with postmenopausal breast cancer (C50 for women 50 years or older). Why were all women included?

**Authors:** In the new version of the paper we analyzed information from adult and older adult women in Brazil (aged ≥ 20 years) without stratifying by age. I respect the reviewer's opinion that the WCRF reports that physical activity is related to postmenopausal breast cancer. However, in the present study we used a reference for PAF that provided evidence for the female population over 20 years of age. In addition, the guidelines of physical activity make it clear that there is sufficient evidence of the relationship between physical activity and breast cancer in women adults and older adults (before and after menopause). For this reason, in the present study we made this methodological decision. The following paragraph was added to the discussion section: “There is a lot of debate in the literature about the effect of physical activity on the prevention of breast cancer before and after menopause [39-41]. All health agencies reinforce that the effect of physical activity in preventing breast cancer is more evident after menopause. However, before menopause there is a theoretical discussion that still needs consensus. The World Cancer Research Fund report describes that the strongest evidence that exists in the relationship between physical activity and breast cancer prevention is for women after menopause [39]. Physical activity guidelines describe that there is strong evidence that physical activity prevents breast cancer before and after menopause [40, 41]. The present study adopted the recommendations of the physical activity guidelines because they were the most recent on the subject. In addition, in the present study was used a reference for PAF that which investigated adult and older adults women [24]”.

**Reviewer #4:**

6. The methodology section needs to be better organized and written. The suggestion is to divide the section into four subsections: study design, hospital cost data, RR and prevalence of physical inactivity, and data analysis. The study design is a cost-of-illness study to estimate the direct costs of breast cancers attributable to lack of physical activity from the perspective of the Brazilian SUS. This approach uses aggregated disease costs, and attributable population fraction (PAF) estimates to calculate the costs attributable to a given risk factor. Hospital cost data should inform the data source and how they were collected (this information is already contained in the latest version of the manuscript). The RR and prevalence subsection should inform where the values were taken from. In the case of prevalence, the data source, the eligible population, and how the METS of the selected population were calculated need to be precise. Data analysis should inform how the PAF (equation) was calculated, the variables included in the equation, how the PAF was applied to expenses, and which software was used to calculate the final amounts.

**Authors:** The change was made.

**Reviewer #4:**

7. Results must prioritize spending.

**Authors:** In the new version of the paper we prioritize spending information.

**Reviewer #4:**

8. The reason for separating expenditures into 20-59 years and ≥ 60 years does not seem adequate. There are no restrictions on physical activity for those over 65s in the latest version of the WHO physical activity guide (2020). Therefore, any policy, program, or action involving physical activity will have the same recommendation regardless of premenopausal or postmenopausal women.

It is appropriate to analyze spending only in postmenopausal women since, according to WCRF, physical activity is related to postmenopausal breast cancer.

**Authors:** In the new version of the paper we analyzed information from adult and older adult women in Brazil (aged ≥ 20 years) without stratifying by age. I respect the reviewer's opinion that the WCRF reports that physical activity is related to postmenopausal breast cancer. However, in the present study we used a reference for PAF that provided evidence for the female population over 20 years of age. In addition, the guidelines of physical activity make it clear that there is sufficient evidence of the relationship between physical activity and breast cancer in women adults and older adults (before and after menopause). For this reason, in the present study we made this methodological decision. The following paragraph was added to the discussion section: “There is a lot of debate in the literature about the effect of physical activity on the prevention of breast cancer before and after menopause [39-41]. All health agencies reinforce that the effect of physical activity in preventing breast cancer is more evident after menopause. However, before menopause there is a theoretical discussion that still needs consensus. The World Cancer Research Fund report describes that the strongest evidence that exists in the relationship between physical activity and breast cancer prevention is for women after menopause [39]. Physical activity guidelines describe that there is strong evidence that physical activity prevents breast cancer before and after menopause [40, 41]. The present study adopted the recommendations of the physical activity guidelines because they were the most recent on the subject. In addition, in the present study was used a reference for PAF that which investigated adult and older adults women [24]”.

9. The discussion and conclusion sections should be rewritten in light of the above recommendations.

**Authors:** We really appreciate your comments on the article.
