# Supplementary material for: Hospitalization and ambulatory costs related to breast cancer due to physical inactivity in the Brazilian state capitals
Source: PLoS One. 2022 Jan 19;17(1):e0261019. doi: 10.1371/journal.pone.0261019 (PMC8769291; doi:10.1371/journal.pone.0261019)
Supplement: Attachment — Submitted filename: review II.doc [file pone.0261019.s007.doc]

1. Recommendation (Answer options: **Major Revision**).

- *It is very limited to a single sentence. It should address the responsible body for the recommended project. It should also be recommended based on the finding.*

2. Is the manuscript technically sound, and do the data support the conclusions? (Answer options: **Partly**).

- *The conclusion should not be a summary, It is a copy past of the main finding.*

3. Has the statistical analysis been performed appropriately and rigorously? (Answer options: **No**).

- *The analysis focus only on descriptive part. It ignored the higher order statistics.*

4. Does the manuscript adhere to the PLOS Data Policy? Additional details can be found at <http://journals.plos.org/plosone/s/materials-and-software-sharing>. (Answer options: **Yes)**
5. Is the manuscript presented in an intelligible fashion and written in standard English? (Answer options: **Yes**)
6. Review Comments to the Author (minimum 100 characters).

- *The topic is of great public health importance. But it only focused on description of the situation. It should be very informative to the reader and policy makers. So, higher order statistics should be included. Sound study participants categorization should be include.*

7. PLOS authors have the option to publish the peer review history of their article

([https://journals.plos.org/plosone/s/editorial-and-peer-review-process#loc-peer-review-history](https://journals.plos.org/plosone/s/editorial-and-peer-review-process#loc-peer-review-history)). If published, this will include your full peer review and any attached files. Do you want your identity to be public for this peer review? (Answer options: **No**)
7a. If you answered "Yes" above, that you would like your identity to be public, please provide your full name, as it should appear on the published peer review. Please do not sign the review on behalf of another person.

Confidential to Editor:
1. Do you have any potentially competing interests? "**None."** Our policy on competing interests can be found at <http://journals.plos.org/plosone/s/competing-interests>.
2. Did you receive any assistance in preparing this review (e.g. from a post-doc or graduate student)? **No.** If yes, please provide their name and title. If you have chosen to reveal your identity to the authors in question 7 above, please include the name of the person who assisted you in the response to question 7 as well. (optional)
