# Supplementary material for: Hospitalization and ambulatory costs related to breast cancer due to physical inactivity in the Brazilian state capitals
Source: PLoS One. 2022 Jan 19;17(1):e0261019. doi: 10.1371/journal.pone.0261019 (PMC8769291; doi:10.1371/journal.pone.0261019)
Supplement: Attachment — Submitted filename: Response to Reviewers 5.doc [file pone.0261019.s008.doc]

**Reviewer #4:**

I have only a final commentary. The author said that periodic exams prevent breast cancer (lines 315-316): "it is necessary that cities invest in other initiatives for the prevention of breast cancer, such as periodic exams." It's not true. Periodic exams such as mammography, contrary to routine cervical cancer exams that lower cervical cancer cases, increase the number of breast cancers.

**Authors:** We really appreciate your comments. In the new version of the article we have removed that sentence.

**Reviewer #7:**

The topic is of great public health importance. But it only focused on description of the situation. It should be very informative to the reader and policy makers. So, higher order statistics should be included. Sound study participants categorization should be include.

**Authors:** We really appreciate your comments. This is a cost-of-illness study to estimate the direct costs of breast cancers attributable to lack of physical activity from the perspective of the Brazilian health services. The study's analysis units are the states of Brazil. Thus, in the article we are not working with data from individuals, but with data from the states of Brazil. In the new version of the article, we've added this information more explicitly**: “The study's analysis units are the states of Brazil”**.
